# Emotions and Sport Management: A Bibliometric Overview

**DOI:** 10.3389/fpsyg.2020.01512

**Published:** 2020-07-10

**Authors:** Hugo Baier-Fuentes, María Huertas González-Serrano, Manuel Alonso-Dos Santos, Williams Inzunza-Mendoza, Victor Pozo-Estrada

**Affiliations:** ^1^Department of Business Administration, Universidad Católica de la Santísima Concepción, Concepción, Chile; ^2^Department of Teaching and Learning of Physical Education, Plastic and Music Education, Universidad Católica de Valencia, Valencia, Spain; ^3^Department of Marketing and Market Research, Facultad de Ciencias Económicas y Empresariales, University of Granada, Granada, Spain

**Keywords:** emotions, sport management, bibliometric analysis, h-index, mapping science

## Abstract

Emotions are considered a fundamental aspect of sport scenarios, and within sports, consumer behavior is a very popular area of research in the sport management field. Thus, in recent years, there has been a growing interest for sport managers regarding the role that emotions play in sport consumer behavior. Thus, the aim of this paper is to provide an overview of the academic research on emotions in the sport management field using two techniques: a bibliometric performance analysis and a graphic mapping of the references in this field. This analysis focuses on authors, journals, papers, institutions and countries. Bibliometric indicators including the h-index measure, productivity and the number of citations were used to perform the performance analysis. Then, VOSviewer software was used to perform co-citation, bibliographic coupling and co-occurrence of keyword analysis (mapping analysis). The results of both types of analysis are consistent, with the United States being the most influential country in emotions in sport management research because the main authors and institutions in this research field belong to this country. The overall results indicate that the literature on this research topic has grown significantly in recent years in all scientific disciplines; however, the research topic is incipient, and therefore, the number of articles is still limited. Thus, this research presents the key aspects in the topic of emotions in sport management that could be helpful for researchers and policy makers in the field of sport management to make future decisions.

## Introduction

Emotions are considered an important component of the client’s experience (Schmitt, 1999) and have become a fundamental aspect of sport scenarios in recent years (Pedragosa et al., 2015). Numerous studies have pointed out how emotions influence the performance of sportsmen and sportswomen (e.g., Jones, 2003; Campo et al., 2019; van Kleef et al., 2019). Although the literature on the role of emotions in the sport business was very limited until a few years ago (Wolfe et al., 2005), it has currently become the central axis of the sport business in contemporary societies (Rodriguez-Pomeda et al., 2017). In fact, the sport consumer has become a popular area of study for sport management researchers (Funk et al., 2016) in which the importance of consumer satisfaction and emotions to enhance loyalty has been highlighted (Koenig-Lewis and Palmer, 2014). This focus has developed because knowing customers’ emotions and the way they express them allows sport managers to act on these emotions and reproduce them in an efficient way, with the aim of influencing and guiding them for the benefit of the organization (Puig, 2012).

Emotions can be defined as a set of interactions between objective and subjective factors influenced by neuronal and hormonal systems that can generate affective experiences such as feelings of activation and liking or disliking, cognitive processes such as perceptions and evaluations, the activation of physiological processes and behavior that is general (Kleinginna and Kleinginna, 1981). In the same vein, Scherer (Scherer, 1987, p. 7) defines emotions as “a sequence of interrelated, synchronized changes in the states of all five organismic subsystems in response to the evaluation of an external or internal stimulus event as relevant to central concerns of the organism.” However, the definition of emotion is ambiguous (Vallerand and Blanchard, 2000), and despite recognizing the central role of emotions in consumer behavior, there is still no agreement on its definition (Carneiro et al., 2019). However, it can be seen how emotions are a key factor in general human behavior (Lewis et al., 2008) and in sport consumer behavior (Silla et al., 2014; Calabuig et al., 2015; Alonso-Dos Santos et al., 2019).

Therefore, research on the link between emotions and post-purchase reactions is essential to helping club managers develop strategies to increase spectator attendance at sport events and improve customer retention (Biscaia et al., 2012). In fact, emotions are one of the main reasons for attending a sport event (Malchrowicz-Mośko and Chlebosz, 2019), and specific studies have been conducted on how specific emotions contribute to the increased satisfaction and behavioral intentions of spectators during sport events (Biscaia et al., 2012; Calabuig et al., 2016; Jang et al., 2019). Likewise, within the theme of sport events, more recent studies have also analyzed the role of volunteers’ emotions in their participation in sport events (Gellweiler et al., 2019). This focus has developed because knowing the emotions within the volunteer population is interesting since volunteering has become increasingly important and is an integral part of the successful organization of sport events and activities (e.g., Dickson et al., 2014; Güntert et al., 2015; Benson and Wise, 2017).

On the other hand, emotions have also been studied within both public and private sport services (Silla et al., 2014; Pedragosa et al., 2015; Ong and Yap, 2017; Foroughi et al., 2019). According to various authors (Dubé and Menon, 2000), users experience emotions during the consumption or practice of activities in a sports center, which arise not only from internal or external attributions but also from interaction with employees or monitors, thereby influencing the final outcome of the experience. Therefore, it can be seen that emotions are important in the field of marketing because emotions can generate differentiation between products and brands through experiences and sensations that arouse these emotions in the consumer (Alvarado, 2008). However, no studies have analyzed the evolution of this area of study of sport management.

Bibliometrics or scientometric analysis is the area of research that helps to analyze current trends in the literature within a particular area and provides guidelines and motivation for future research (Muhuri et al., 2019). Bibliometric analysis can provide more objective and comprehensive results than typical literature reviews (Ramos-Rodrígue and Ruíz-Navarro, 2004). This type of study is common in a wide range of journals, such as *Journal of Knowledge Management* (Gaviria-Marin et al., 2018, 2019), *International Business Review* (Rialp et al., 2019) or *Computers & Industrial Engineering* (Cancino et al., 2019), to name a few. Although they are also usual to study and analyze specific research fields, such as knowledge management (Gaviria-Marin et al., 2019), international entrepreneurship (Baier-Fuentes et al., 2019), sport entrepreneurship (González-Serrano et al., 2019), sport management (Ciomaga, 2013), sport management and educational management (Belfiore et al., 2019), and fitness equipment (Addolorato et al., 2019). However, no studies that focus on emotions within the field of sport management have been found to the best of our knowledge. Therefore, based on the background presented, the main aim of this paper is to provide a broad quantitative and qualitative view of emotions in the sport management field (ESM hereinafter) by using performance analysis and science mapping. Articles from the journals indexed in Scopus will be analyzed because Scopus is considered one of the most complete databases in the social sciences (Mongeon and Paul-Hus, 2016). The analyses are performed considering the following information: years, authors, papers, journals, institutions and countries. The references were obtained considering all the documents published between 1989 and 2019 in the Scopus database.

The results of the bibliometric analysis, both for the performance analysis and the graphic mapping, are consistent among the two techniques and show that Kaplanidou and Madrigal are among the most productive and influential author regarding emotions in the field of sport management research. Other leaders in the field who are also in top positions are Smith, and more recently, Filo, Calabuig and Crespo, who are gaining importance. The journals focused on sport and service management are the most productive and influential. Among them, the *Sport Management Review* was found to be the most influential journal, followed by, the *European Sport Management Quarterly, Journal of Services Marketing* and the *Journal of Service Research*. The United States led the research of emotions in sport management (followed by the Australia, United Kingdom, Germany and Canada) because it hosted the most influential authors and institutions in this field of research. However, in the last five years, other countries such as Spain and South Korea, for example, have contributed significantly to the productivity on this topic, although they are still far from the top positions in the field. Finally, it is important to highlight that the consistency of the results obtained from the two bibliometric approaches provides valuable information within the field of emotions and sport management.

Thus, this paper is organized as follows. The relevance of this topic is presented in this section (section “Introduction”). In the following section, the methodology used in this study is presented (section “Materials and Methods”). Then, the results of this study obtained by the performance bibliometric analysis and the bibliometric mapping are presented (section “Results”). Finally, in section “Conclusion,” the main conclusions of this study are presented.

## Materials and Methods

Bibliometrics is a field of research that quantitatively studies bibliographical references (Broadus, 1987); it has become a useful and important technique since it provides general information about the various actors that publish in a particular field of research. In this same line, the usefulness of bibliometric studies lies in finding new research gaps, finding influences or following the research path of a particular scientific actor, among other information.

To find the bibliographic references at the intersection of emotions, sport and management, the Scopus database was used. Among others, Scopus is one of the most important databases among the scientific community, since it has been designed both for the search of bibliographic material and for the analysis of citations (Meho and Yang, 2007), thereby offering the same analysis tools as other frequently used databases such as the Web of Science (WoS hereinafter) (Baier-Fuentes et al., 2019). This database is part of the objective of analyzing the main trends in an area of incipient scientific intersection, such as emotions, sports and management. Therefore, Scopus was selected due that includes the majority of journals indexed in WoS and that it has a greater number of journals (and therefore references) compared to this database (Mongeon and Paul-Hus, 2016). In fact, almost 84% of the articles of WoS can be found in Scopus, and the WoS database includes fewer indexed journals than Scopus, so by selecting Scopus the risk of overlooking documents during the search is reduced (Terán-Yépez et al., 2020). Thus, the reference search was limited to publications found in Scopus and made in the last 30 years (i.e., between 1989 and 2019), whose references were obtained after applying the following keyword combination: [(“emotion^∗^”) AND (“sport^∗^”) OR (“emotion^∗^”) AND (“athletic^∗^”)] AND [(“entrepreneur^∗^”) OR (“sport^∗^ entrepreneur^∗^”) OR (“marketing^∗^”) OR (“sport^∗^ marketing^∗^”) OR (“management^∗^”) OR (“sport^∗^ management”) OR (“sport^∗^ event^∗^”) OR (“athleti^∗^ event^∗^”)]. Subsequently, given the objective of this work, references were selected from the areas of business, management, accounting, economics, sociology, psychology, decision sciences and neuroscience. In addition, to analyze only research studies, only the articles, reviews, notes and letters were selected (Merigó et al., 2015), obtaining a total of 353 references. These references were thoroughly reviewed and only those focusing on the intersection of Emotions and Sport Management were selected, namely 153 references.

According to Noyons et al. (1999), bibliometric studies can involve various complementary methodological techniques, such as performance analysis and the graphic mapping of science, which is also known as bibliometric mapping (Cobo et al., 2010). The first of these techniques uses several bibliometric indicators known in the scientific community. However, there is a controversy in the literature about what indicator could better measure scientific production (Podsakoff et al., 2008). Among the most popular indicators are the number of publications and the number of citations, which represent productivity and influence, respectively (Baier-Fuentes et al., 2019). Some researchers, however, criticize the number of articles indicator, because a large number of publications does not imply a greater impact or quality of academic research. Similarly, the number of citations has also received criticism, since an author with a high number of citations does not imply more or less quality in his or her research (Cancino et al., 2017b). This is due, among other things, to the author’s area of study, in which he may receive a greater or lesser number of citations (Bonilla et al., 2015). The h-index was also used, which integrates in a single measure the number of publications with the number of citations (Hirsch, 2005). This indicator is easy to interpret and indicates the number of N studies that have received at least N citations. However, despite its easy interpretation, this index has not been exempting from criticism. In this sense, Ye and Leydesdorff (2014) points out that the h-index only increases with time, allowing the author to rest confident in this indicator. It has also been noted that this index does not take into account highly cited documents; that is, it ignores citations received above the level of the index. Conversely, it has been noted that the index ignores documents that have not reached the number of citations in h-index (Zhang, 2013; Egghe, 2010). These limitations or criticisms have triggered the emergence of several other indicators such as g-index (Egghe, 2006), AR-index (Jin et al., 2007), hg-index (Alonso et al., 2009), p-index (Prathap, 2010), among several others. However, some studies such as Yan et al. (2016), Cancino et al. (2017a), show that there are no major differences between these indicators, with the exception of some that are little used. Also, although any one of them could be used, the fact is that all indicators have limitations in measuring bibliometric performance (Agarwal et al., 2016). Indeed, Ding et al. (2020) point out that the limitations of these indicators complicate the appropriate choice of indicator to assess research performance. Therefore, and in order to use indicators that are easy to interpret, and also known by the scientific community, in this study we used the h-index. Other indicators that are used are the index of citations per article, citation thresholds, which measure the number of articles over a specific number of citations; and temporality analyses, which allow the analyze the publication behavior of the scientific actors, namely, journals, articles, authors, institutions and countries.

On the other hand, the graphic mapping of science focuses on showing the intellectual connections between the scientific actors who work in a specific field and who are thus related to each other. This graphical representation is obtained from a scientific repository that changes frequently over time (Cobo et al., 2011b) and has been strengthened thanks to computer advances in the development of software that allow the analysis of references (Cobo et al., 2011b). Among the most popular software in the scientific community are BibExcel (Persson et al., 2009), CiteSpace II (Chen, 2006), IN-SPIRE (Wise, 1999), Vantage Point (Porter and Cunningham, 2005), and VOSviewer (van Eck and Waltman, 2010). Given the experience of the researchers, this study uses the VOSviewer software, which carries out its analyses based on different indicators, such as co-citations (Small, 1973), co-authorships (Peters and van Raan, 1991), bibliographic coupling (Kessler, 1963) and co-words (Callon et al., 1983). Please note that co-citation analyses documents that receive citations from the same third documents, by mapping the most cited sources (represented by the size of the circles), and the connection between those cited by the same sources represented by lines (Cancino et al., 2017b; Valenzuela et al., 2017). Co-authorship measures the degree of co-authorship that has developed in the field of study. Bibliographic coupling measures the number of times that two documents cite the same third document represented the most influential documents (represented by size of the circles) and similarity in the reference profile (Kessler, 1963). Finally, co-occurrence of keywords is used to study the conceptual structure of the field of study and to know - given the size of the circles - the keywords most frequently used in documents (Laengle et al., 2017; Martínez-López et al., 2018). This analysis is also used to know the conceptual evolution of the field of study over time. In the figure, the thickness of the lines represents the intensity or the strength of the link between the analyzed elements, hence, the thicker the line is, the higher the number of co-occurrences between these elements (Calabuig-Moreno et al., 2020).

Therefore, based on the developed background, the present article includes a complete performance analysis and the graphic mapping of the field of research in an updated state. However, given the changing dynamics of science, the data presented may change over time, especially for those more recent publications that, naturally and depending on their quality, must improve their indicators.

## Results

### Performance Bibliometric Analysis

This section presents the main results of the bibliometric performance analysis. For this purpose, the series of indicators described above will be taken into account based on the number of articles published, the number of citations received, and the h-index, among others.

First, the general aspects of the research topic are shown. This research analyses the publications related to this topic from 1989 onward. It must be taken into account that the research topic is incipient, and therefore, the number of articles is still limited. In addition, as is logical, the references are distributed throughout different scientific disciplines, as shown in Table 1.

**TABLE 1:** Research Area in research topic.

R	Area	TP
1	Medicine	240
2	Business, Management, and Accounting	185
3	Social Sciences	140
4	Psychology	121
5	Health Professions	110
6	Computer Science	61
7	Decision Sciences	49
8	Engineering	35
9	Arts and Humanities	33
10	Neuroscience	27
11	Economics, Econometrics and Finance	26
12	Nursing	20
13	Biochemistry, Genetics and Molecular Biology	19
14	Agricultural and Biological Sciences	15
15	Environmental Science	15
16	Mathematics	15
17	Materials Science	11
18	Energy	7
19	Multidisciplinary	7
20	Physics and Astronomy	6
21	Chemical Engineering	3
22	Earth and Planetary Sciences	3
23	Veterinary	3
24	Chemistry	2
25	Pharmacology, Toxicology and Pharmaceutics	2
26	Dentistry	1
27	Immunology and Microbiology	1
28	Undefined	1

*Source: Own elaboration based on Scopus 2019. TP, Total Papers.*

**FIGURE 1:**
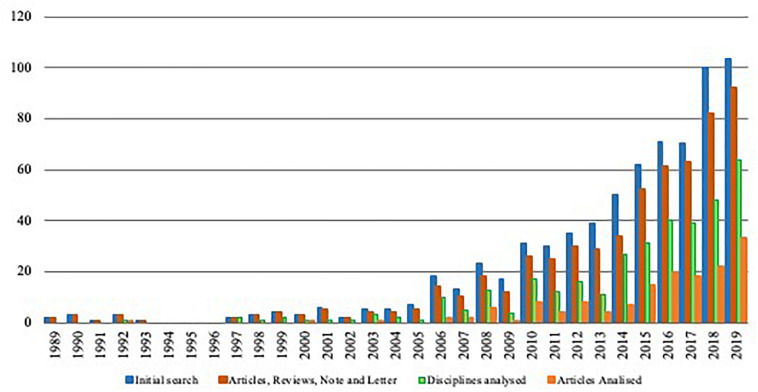
Growth trend in research topic publications.

Likewise, Figure 1 shows the growth trend of publications made at the intersection of the above disciplines. Note that the blue bars represent the 709 references that are somehow related to the research topic. These bars include all types of documents, such as articles, conference papers, reviews, book chapters, and books. However, it is part of the bibliometric methodological procedure to analyze only research papers, namely, articles, reviews, notes and letters. Therefore, the red bars in the figure represent these types of documents. The green bars represent the references that converge on the research topics. However, the orange bars represent the references that are the final objective of our study, which is to analyze the literature at the intersection of emotions and sports management.

Another way to analyze the growth and the influence of the literature on this research topic is through the general citation structure of the publications, as presented in Table 2. The publications are ordered according to the thresholds of citations received and the year they were published. Other general indicators corresponding to each year are also included. It can be seen that until 2006, the growth of the literature was slow, and its influence was not significant. Since 2006, the literature has been characterized by slow growth, but some articles are published that, to date, are the most influential on this topic. For example, the year 2008 is important because Martin et al., published his article “The role of emotion in explaining consumer satisfaction and future behavioral intention” in the *Journal of Sport Management*. This article is the most cited on this topic, with 118 citations, and it focuses on the emotions of the match spectators of a football stadium. A time-elapsed three-stage survey was used to evaluate the changes of emotions over time. Finally, these authors highlight the need to use emotional and cognitive measures of satisfaction to measure and evaluate customer satisfaction and future behavioral intention of sport spectators.

**TABLE 2:** Structure of research topic citations.

Year	TPES	TCES	HES	ACES	%PES	≥100	≥50	≥25	≥10	≥5
1992	1	19	1	19,00	0,01	–	–	–	1	–
1993	–	–	–	–	–	–	–	–	–	–
1994	–	–	–	–	–	–	–	–	–	–
1995	–	–	–	–	–	–	–	–	–	–
1996	–	–	–	–	–	–	–	–	–	–
1997	–	–	–	–	–	–	–	–	–	–
1998	–	–	–	–	–	–	–	–	–	–
1999	–	–	–	–	–	–	–	–	–	–
2000	1	54	1	54,00	0,01	–	1	–	–	–
2001	–	–	–	–	–	–	–	–	–	–
2002	–	–	–	–	–	–	–	–	–	–
2003	1	59	1	59,00	0,01	–	1	–	–	–
2004	–	–	–	–	–	–	–	–	–	–
2005	–	–	–	–	–	–	–	–	–	–
2006	2	70	2	35,00	0,01	–	1	–	1	–
2007	2	98	2	49,00	0,01	–	1	1	–	–
2008	6	301	5	50,17	0,04	1	–	3	–	1
2009	1	25	1	25,00	0,01	–	–	1	–	–
2010	8	258	7	32,25	0,05	–	1	3	2	–
2011	4	85	4	21,25	0,03	–	–	2	1	1
2012	8	294	7	36,75	0,05	–	3	1	2	1
2013	4	44	3	11,00	0,03	–	–	1	1	–
2014	7	26	3	3,71	0,05	–	–	–	1	1
2015	15	196	7	13,07	0,10	–	1	1	4	2
2016	20	181	7	9,05	0,13	–	–	3	3	5
2017	18	95	6	5,28	0,12	–	–	–	4	3
2018	22	72	5	3,27	0,14	–	–	–	–	6
2019	33	37	4	1,12	0,22	–	–	–	1	–

*Source: Own elaboration based on Scopus 2019. Abbreviations: R, Ranking; TCES and TPES, total citations and papers only ESM field; HES, h-index only ESM field; ACES, average citations per paper;%PES, percentage of papers compared to the total of papers in the ESM field; ≥100, ≥50, ≥25, ≥10, ≥5 number of paper with more than 100, 50, 25, 10, and 5 citations.*

In the following decade (2006−2019), 98% of the total articles analyzed in this study were published, of which 30% received at least 10 citations. In summary, given the incipient nature of this research topic, there are still articles to be positioned in order to set trends and influence the research topic. To date, only two articles have received more than 100 citations, but it is hoped that other articles of a theoretical nature will be able to provide influence and mark out clear paths of research in these areas.

#### The Most Influential Journal in ESM Research

As mentioned, the articles published at the intersection of emotions and sport management are studied in several disciplines, and logically, a wide range of journals, including some that specialize in this area, have published articles on this topic. Table 3 shows the classification of the 40 most influential and productive journals in this field. Other indicators concerning the evolution of publications by decade or the citation thresholds of the articles published by each journal have been included in this table. In addition, other general indicators of the journals, such as the total number of articles published, the total citations and the h-index, are also presented. Finally, the journals are ordered according to their influence on the research topic (TCES *hereinafter*). In case of a tie in the number of citations, the total number of publications is considered (TPES *hereinafter*), followed by the h-index (HES *hereinafter*). These three indicators (TCES, TPES and HES) consider only the number of citations, papers and the h-index of each paper in the ESM research topic. Note that general indicators of the journal are presented with respect to the number of citations (TC), number of papers (TP) and the h-index (H).

**TABLE 3:** Most influential journal in ESM research.

R	Journal	TCES	TPES	HES	%PES	≥100	≥50	≥25	≥10	≥5	D1	D2	D3	2019	TP	TC	SJR	T50	H
1	SMR	242	20	8	13,07	–	1	3	2	3	–	1	10	9	620	11.185	1.769	6	50
2	ESMQ	155	7	4	4,58	–	2	1	1	–	–	–	4	3	327	3.281	1.280	4	29
3	JSMk	134	2	2	1,31	1	–	1	–	–	–	1	1	–	1.316	45.397	1.021	2	102
4	JSR	122	3	3	1,96	–	1	1	1	–	–	2	1	–	621	49.660	3.340	3	113
5	JSM	99	6	5	3,92	–	1	–	3	1	–	–	6	–	684	15.711	1469	2	61
6	EM	93	6	3	3,92	–	1	–	1	–	–	1	3	2	502	3.876	0.488	2	30
7	JLR	84	1	1	0,65	–	1	–	–	–	–	1	–	–	852	24.957	0.53	1	74
8	CHB	81	2	2	1,31	–	1	–	1	–	–	–	1	1	6.382	149.856	1.711	2	137
9	AMA	80	1	1	0,65	–	1	–	–	–	–	–	1	–	182	15.463	12.7	1	66
10	IJSMS	78	4	3	2,61	–	–	2	1	–	–	–	3	1	326	1.725	0.419	3	20
11	JST	55	6	3	3,92	–	–	1	2	–	–	–	3	2	425	6.002	0.581	3	40
12	JBR	32	3	2	1,96	–	–	1	–	–	–	–	2	1	6.741	180.021	1684	1	158
13	JCP	32	1	1	0,65	–	–	1	–	–	–	1	–	–	1.172	51.671	2.98	1	113
14	IJHM	31	1	1	0,65	–	–	1	–	–	–	–	1	–	4.866	13.257	2	1	93
15	JN	29	1	1	0,65	–	–	1	–	–	–	–	1	–	36.695	635.006	4.21	1	422
16	S&S	25	3	3	1,96	–	–	–	2	–	–	–	3	–	711	3.399	0.403	2	24
17	APJML	25	3	3	1,96	–	–	–	1	1	–	–	3	–	986	9.604	0.333	1	41
18	ETP	24	1	1	0,65	–	–	–	1	–	–	–	1	–	943	72.450	5.07	1	146
19	CPD	20	3	3	1,96	–	–	–	1	–	–	–	3	–	462	1.560	0.379	1	14
20	IJESB	20	1	1	0,65	–	–	–	1	–	–	–	1	–	1.419	11.205	0.4	1	39
21	HBR	19	1	1	0,65	–	–	–	1	–	1	–	–	–	4.268	20.153	0.22	1	161
22	C&S	16	4	3	2,61	–	–	–	–	2	–	–	4	–	210	821	0.721	–	14
23	IJEFM	15	2	1	1,31	–	–	–	1	–	–	–	1	1	206	1.758	0.445	1	22
24	SMQ	10	2	2	1,31	–	–	–	–	1	–	–	2	–	43	93	0.2	–	5
25	SBMIJ	9	3	2	1,96	–	–	–	–	1	–	–	3	–	207	844	0.278	–	14
26	IJSMM	7	5	2	3,27	–	–	–	–	–	–	1	4	–	384	2.119	0.263	–	21
27	SBP	7	3	2	1,96	–	–	–	–	–	–	–	3	–	2.580	3.731	0.279	–	47
28	JVM	7	1	1	0,65	–	–	–	–	1	–	–	1	–	741	16.362	0.99	–	62
29	JCET	6	2	1	1,31	–	–	–	–	1	–	–	1	1	299	2.420	0.266	–	24
30	EE	6	1	1	0,65	–	–	–	–	1	–	–	1	–	651	4.777	0.288	–	27
31	INN	6	1	1	0,65	–	–	–	–	1	–	1	–	–	583	1.036	0.16	–	10
32	LS	6	1	1	0,65	–	–	–	–	1	–	–	1	–	1.126	20.854	0.74	–	69
33	SARSPER	5	2	1	1,31	–	–	–	–	1	–	–	2	–	401	1.034	0.19	–	13
34	JHTM	5	1	1	0,65	–	–	–	–	1	–	–	1	–	451	4.289	0.82	–	30
35	JPBM	5	1	1	0,65	–	–	–	–	1	–	–	1	–	815	12.891	0.86	–	82
36	FP	4	1	1	0,65	–	–	–	–	–	–	–	1	–	14.252	117.161	0.977	–	94
37	SiS	3	2	1	1,31	–	–	–	–	–	–	–	1	1	1.602	9.965	0.55	–	37
38	JFMM	3	1	1	0,65	–	–	–	–	–	–	–	1	–	762	10.577	0.653	–	47
39	JGFM	3	1	1	0,65	–	–	–	–	–	–	–	1	–	225	1.494	0.21	–	18
40	JGSM	2	2	1	1,30719	–	–	–	–	–	–	–	2	–	133	209	−−	–	6

*R, Ranking; TCES and TPES, total citations and papers only ESM field; HES, h-index only ESM field;%PES, percentage of ESM papers in the journal; T50, number of papers in the top 50 list shown in Table 4; ≥100, ≥50, ≥25, ≥10, ≥5 number of paper with more than 100, 50, 25, 10, and 5 citations; D1: 1989−1998; D2: 1999−2008; D3: 2009−2018. H, h-index of journal; TP and TC, total papers and citations; SJR, SCImago Journal Rank measures − Scopus 2019. SMR, Sport Management Review; ESMQ, European Sport Management Quarterly; JSMk, Journal Of Services Marketing; JSR, Journal Of Service Research; JSM, Journal Of Sport Management; EM, Event Management; JLR, Journal of Leisure Research; CHB, Computers In Human Behavior; AMA, Academy Of Management Annals; IJSMS, International Journal Of Sports Marketing And Sponsorship; JST, Journal Of Sport And Tourism; JBR, Journal Of Business Research; JCP, Journal of Consumer Psychology; IJHM, International Journal of Hospitality Management; JN, Journal Of Neuroscience; S&S, Soccer And Society; APJML, Asia Pacific Journal Of Marketing And Logistics; ETP, Entrepreneurship: Theory and Practice; CDP, Cuadernos De Psicologia Del Deporte; IJESB, International Journal of Entrepreneurship and Small Business; HBR, Harvard Business Review; C&S, Communication And Sport; IJEFM, International Journal Of Event And Festival Management; SMQ, Sport Marketing Quarterly; IJSMM, International Journal Of Sport Management And Marketing; SBP, Social Behavior And Personality; JVM, Journal of Vacation Marketing; JCET, Journal Of Convention And Event Tourism; EE, Engineering Economics; INN, Innovar; LS, Leisure Studies; SARSPER, South African Journal For Research In Sport Physical Education And Recreation; JHTM, Journal of Hospitality and Tourism Management; JPBM, Journal of Product and Brand Management; FPs, Frontiers In Psychology; SiS, Sport in Society; JFMM, Journal Of Fashion Marketing And Management; JGFM, Journal of Global Fashion Marketing; JGSM, Journal Of Global Sport Management.*

According to Table 3, the most influential journal in the ESM research topic is the *Sport Management Review* (SMR), with 242 citations. Note that 98 of these quotes are those received by the 2010 Smith and Stewart article. Other authors who have contributed to the positioning of this journal are Lamont, Hing and Vitartas or Doyle, Filo, Funk and McDonald with their 2016 articles which have each received 26 citations. *Sport Management Review* is also the most productive journal, with 20 articles, representing 13.1% of the articles published on this topic. Other influential journals that appear at the top of the ranking are specific journals in the field of Sports Management and the area of Business or Marketing, such as the *European Sport Management Quarterly* or the *Journal of Sport Management*, as well as the *Journal of Service Marke*ting or the *Journal of Service Research*. Finally, from a more general perspective, the results show that research on this topic has been progressively published in various journals. In fact, the last decade has been very productive, and almost all journals published at least one paper in this field of research. Even so, it should be noted that despite an increase in research, several journals have stopped publishing on this topic. Therefore, it is expected that other journals will take an interest in this interesting research topic.

#### The Most Influential Articles in ESM Research

Another aspect that is interesting to analyze is the most influential publications on this research topic. That is, those that have received the most citations. Note that the number of citations (TC in Table 4), is a reflection of the popularity and influence that each article has in the scientific community (Baier-Fuentes et al., 2019). Table 4 presents the 50 most cited articles in this research topic.

**TABLE 4:** The most cited papers in topic research.

R	Title	Authors	Year	Journal	TCES	C/Y
1	The role of emotion in explaining consumer satisfaction and future behavioral intention	Martin, D., O’Neill, M., Hubbard, S., Palmer, A.	2008	JSM	118	9,8
2	The special features of sport: A critical revisit	Smith, A. C. T., Stewart, B.	2010	SMR	98	9,8
3	Investigating an evolving leisure experience: Antecedents and consequences of spectator affect during a live sporting event	Madrigal, R.	2003	JLR	84	4,9
4	The Sporting Life: Exploring Organizations through the Lens of Sport	Day, D. V., Gordon, S., Fink, C.	2012	AMA	80	10,0
5	How social media engagement leads to sports channel loyalty: Mediating roles of social presence and channel commitment	Lim, J. S., Hwang, Y., Kim, S., Biocca, F. A.	2015	CHB	71	14,2
6	Affective event and destination image: Their influence on olympic traveler behavioral intentions	Kaplanidou, K.	2007	EM	71	5,5
7	Consumer orientation toward sporting events: Scale development and validation	Pons, F., Mourali, M., Nyeck, S.	2006	JSR	59	4,2
8	The effects of emotions on football spectators’ satisfaction and behavioral intentions	Biscaia, R., Correia, A., Rosado, A., Maroco, J., Ross, S.	2012	ESMQ	56	7,0
9	The importance of legacy outcomes for Olympic Games four summer host cities residents’ quality of life: 1996−2008	Kaplanidou, K.	2012	ESMQ	55	6,9
10	The meaning and measurement of a sport event experience among active sport tourists	Kaplanidou, K., Vogt, C.	2010	JSM	54	5,4
11	Addressing participation constraint: A case study of potential skiers	Williams, P., Fidgeon, P. R.	2000	TM	54	2,7
12	Gendered managerial discourses in sport organizations: Multiplicity and complexity	Knoppers, A., Anthonissen, A.	2008	SR	49	4,1
13	The dynamics underlying service firm-customer relationships: Insights from a study of English Premier League soccer fans	Harris, L. C., Ogbonna, E.	2008	JSR	41	3,4
14	Examining the relationship between brand emotion and brand extension among supporters of professional football clubs	Abosag, I., Roper, S., Hind, D.	2012	EJM	40	5,0
15	Event image perceptions among active and passive sports tourists at marathon races	Hallmann, K., Kaplanidou, K., Breuer, C.	2006	IJSMS	37	2,6
16	Hot vs. cold cognitions and consumers’ reactions to sporting event outcomes	Madrigal, R.	2008	JCP	32	2,7
17	An empirical model of attendance factors at major sporting events	Hall, J., O’Mahony, B., Vieceli, J.	2010	IJHM	31	3,1
18	Mental hoop diaries: Emotional memories of a college basketball game in rival fans	Botzung, A., Rubin, D. C., Miles, A., Cabeza, R., LaBar, K. S.	2010	JN	29	2,9
19	Affective response to gambling promotions during televised sport: A qualitative analysis	Lamont, M., Hing, N., Vitartas, P.	2016	SMR	28	7,0
20	Spectator emotions: Effects on quality, satisfaction, value, and future intentions	Calabuig, F., Prado-Gascó, V., Crespo, J., Núñez-Pomar, J., Añó, V.	2015	JBR	28	5,6
21	Exploring the role of emotions on sport consumers’ behavioral and cognitive responses to marketing stimuli	Dae, H. K., Yu, K. K., Hirt, E. R.	2011	ESMQ	28	3,1
22	The effect of joy on the behavior of cricket spectators: The mediating role of satisfaction	Kuenzel, S., Yassim, M.	2007	ML	27	2,1
23	Exploring PERMA in spectator sport: Applying positive psychology to examine the individual-level benefits of sport consumption	Doyle, J. P., Filo, K., Lock, D., Funk, D. C., McDonald, H.	2016	SMR	26	6,5
24	Retrospective: the importance of servicescapes in leisure service settings	Wakefield, K. L., Blodgett, J.	2016	JSM	26	6,5
25	Analyzing gender dynamics in sport governance: A new regimes-based approach	Adriaanse, J. A., Schofield, T.	2013	SMR	25	3,6
26	A (mis)guided adventure tourism experience: An autoethnographic analysis of mountaineering in Bolivia	Houge Mackenzie, S., Kerr, J. H.	2012	JST	25	3,1
27	Effects of atmosphere at major sports events: A perspective from environmental psychology	Uhrich, S., Koenigstorfer, J.	2009	IJSMS	25	2,3
28	“Bouncing Back” From a Loss: Entrepreneurial Orientation, Emotions, and Failure Narratives	Wolfe, M. T., Shepherd, D. A.	2015	ETP	24	4,8
29	Spectator Rage as the Dark Side of Engaging Sport Fans: Implications for Services Marketers	Grove, S. J., Pickett, G. M., Jones, S. A., Dorsch, M. J.	2016	JSR	22	5,5
30	Active sport tourists: Sport event image considerations	Kaplanidou, K.	2010	TA	21	2,1
31	Athletes as entrepreneurs: The role of social capital and leadership ability	Ratten, V.	2015	IJESB	20	4,0
32	High-performance marketing: an interview with Nike’s Phil Knight. Interview by Geraldine E. Willigan	Knight, P.	1992	HBR	19	0,7
33	Passion and pride in professional sports: Investigating the role of workplace emotion	Swanson, S., Kent, A.	2017	SMR	18	6,0
34	Impact of core product quality on sport fans’ emotions and behavioral intentions	Foroughi, B., Nikbin, D., Hyun, S. S., Iranmanesh, M.	2016	IJSMS	16	4,0
35	Exploring the positive psychology domains of well-being activated through charity sport event experiences	Filo, K., Coghlan, A.	2016	EM	16	4,0
36	Emotion and memory in nostalgia sport tourism: examining the attraction to postmodern ballparks through an interdisciplinary lens	Gordon, K. O.	2013	JST	15	2,1
37	Images of rural destinations hosting small-scale sport events	Hallmann, K., Breuer, C.	2011	IJEF	15	1,7
38	Sport spectatorship and life satisfaction: A multicountry investigation	Inoue, Y., Sato, M., Filo, K., Du, J., Funk, D. C.	2017	JSM	14	4,7
39	Existence of mixed emotions during consumption of a sporting event: A real-time measure approach	Kim, J. W., Magnusen, M., Lee, H.-W.	2017	JSM	14	4,7
40	Understanding cycle tourism experiences at the Tour Down Under	Shipway, R., King, K., Lee, I. S., Brown, G.	2016	JST	13	3,3
41	Emotions and sponsorship: A key to global effectiveness? A comparative study of Australia and France	Bal, C., Quester, P., Plewa, C.	2010	APJML	13	1,3
42	Evaluation of total quality in sports municipal services geared to children: Contributions from the qualitative analysis ATLAS.ti	Pérez-López, R., Morales-Sánchez, V., Teresa Anguera, M., Hernández-Mendo, A.	2015	CPD	12	2,4
43	Toward emotional quality service oriented sports organizations child population: A qualitative analysis	Pérez-López, R., Morales-Sánchez, V., Teresa Anguera, M., Hernández-Mendo, A.	2015	RIPED	11	2,2
44	Managing dive centers: SCUBA divers’ behavioral intentions	Palau-Saumell, R., Forgas-Coll, S., Sánchez-García, J., Prats-Planagumà, L.	2014	ESMQ	11	1,8
45	Governing by fun: EURO 2008 and the appealing power of fan zones	Lauss, G., Szigetvari, A.	2010	S&S	11	1,1
46	Dropping Out: Why Male and Female Leaders in German Sports Federations Break Off Their Careers	Pfister, G., Radtke, S.	2006	SMR	11	0,8
47	User sentiment analysis based on social network information and its application in consumer reconstruction intention	Zhou, Q., Xu, Z., Yen, N. Y.	2019	CHB	10	10,0
48	Consumers’ perceived value of sport team games-a multidimensional approach	Kunkel, T., Doyle, J. P., Berlin, A.	2017	JSM	10	3,3
49	Fragments of us, fragments of them: social media, nationality and US perceptions of the 2014 FIFA World Cup	Billings, A. C., Burch, L. M., Zimmerman, M. H.	2015	S&S	10	2,00
50	It’s not whether you win or lose; It’s how the game is played:The influence of suspenseful sports programming on advertising	Bee, C., Madrigal, R.	2012	JA	10	1,25

*R, Ranking; Journal abbreviations are available in Table 3; TCES, Total citation of article; C/Y, Citations per year indicator.*

As mentioned previously, the most widely cited research article on this topic was published by Martin et al., 2008 in the *Journal of Sport Management*. Logically, this article is also important for the journal because it has allowed the journal to position itself as one of the five most influential journals on this topic. The second most cited article, with 98 citations, is one by Smith and Stewart, which was published in 2010 in the *Sport Management Review*. Note that both articles show a good indicator of citations per year (9.8 Citation/Year). Related to this indicator of citations per year, an article published in 2015 in *Computer in Human Behavior* written by Lim, Hwang, Kim and Biocca stands out. This article, which receives an average of 14.2 citations per year, reveals that viewers who are able to convey their emotions through a TV sports channel’s social networks during the transmission of a mega sports event such as the 2014 Sochi Olympic Games increase their commitment and loyalty to the TV channel. It is also important to highlight Kaplanidou as the researcher who dominates this list with 5 articles, of which 4 are among the 20 most-cited articles.

#### The Most Productive and Influential Authors in ESM Research

Several authors have contributed to the development of this research topic. In fact, and as is natural, the last few years have seen the emergence of many authors. Table 5 lists the 40 most productive and influential authors in this field of research. Please note that the indicator used to classify these researchers is TCES. In the case of a tie, the indicators considered are TPES and HES.

**TABLE 5:** The most productive and influential authors in ESM research.

R	Name	Country	TCES	TPES	HES	ACP	Q1	Q2	Q3	Q4	TC	TP	H	TP50
1	Kaplanidou, K	USA	240	6	5	40,0	–	1	5	–	1733	58	24	5
2	Madrigal, R	USA	126	3	3	42,0	1	1	1	–	1449	31	15	3
3	Smith, Aaron C. T.	UK	98	2	1	49,0	–	–	1	1	1063	65	19	1
4	Filo, Kevin R.	AUS	56	3	3	18,7	–	–	–	3	708	35	16	–
5	Breuer, C	GER	52	2	2	26,0	–	–	2	–	1424	116	21	2
6	Hallmann, K	GER	52	2	2	26,0	–	–	2	–	904	57	17	2
7	Anthonissen, A	NLD	49	1	1	49,0	–	1	–	–	126	4	4	1
8	Funk, DC.	USA	40	2	2	20,0	–	–	–	2	3570	107	35	2
9	Abosag, I	UK	40	1	1	40,0	–	–	1	–	269	23	9	1
10	Doyle, JP	AUS	36	2	2	18,0	–	–	–	2	177	10	9	2
11	Calabuig-Moreno, F	ESP	34	2	2	17,0	–	–	–	2	391	68	10	1
12	Núñez-Pomar, JM	ESP	34	2	2	17,0	–	–	–	2	139	23	6	1
13	Crespo-Hervás, J	ESP	34	2	2	17,0	–	–	–	2	111	18	6	1
14	Hernández-Mendo, A	ESP	31	4	4	7,8	–	–	–	4	1238	115	19	2
15	Pérez-López, R	ESP	31	4	4	7,8	–	–	–	4	46	5	4	2
16	Lamont, M	AUS	28	2	1	14,0	–	–	–	2	680	43	16	1
17	Anguera-Argilaga, MT	ESP	27	3	3	9,0	–	–	–	3	2319	153	27	2
18	Morales-Sánchez, V	ESP	27	3	3	9,0	–	–	–	3	420	65	13	2
19	Adriaanse, J. A.	AUS	25	1	1	25,0	–	–	1	–	87	10	5	1
20	Kim, JW	USA	18	2	2	9,0	–	–	–	2	58	10	4	2
21	Kent, A	USA	18	2	1	9,0	–	–	–	2	665	26	15	1
22	Swanson, S	UK	18	2	1	9,0	–	–	–	2	66	10	5	1
23	Bee, C	USA	14	2	2	7,0	–	–	1	1	142	12	7	1
24	Kunkel, T	USA	13	2	2	6,5	–	–	–	2	198	19	9	1
25	Ko, Y. J	USA	9	2	2	4,5	–	–	–	2	1397	72	20	–
26	Mutz, M	CHN	8	2	2	4,0	–	–	–	2	187	40	7	–
27	Won, D	USA	8	2	1	4,0	–	–	–	2	238	49	8	–
28	Chiu, W	GER	8	2	1	4,0	–	–	–	2	171	32	7	–
29	Heere, B	USA	7	3	2	2,3	–	–	–	3	761	41	14	–
30	Lee, S	KOR	7	2	2	3,5	–	–	–	2	65	7	3	–
31	Tyler, B.D	USA	6	2	1	3,0	–	–	–	2	111	13	6	1
32	Apostolopoulou, A	USA	6	1	1	6,0	–	–	–	1	216	14	8	–
33	Agha, N	USA	6	1	1	6,0	–	–	–	1	116	12	4	–
34	Byon, K	USA	5	3	1	1,7	–	–	–	3	290	34	7	–
35	Akhoondnejad, A	NZL	5	1	1	5,0	–	–	–	1	57	3	3	–
36	Sato, S	USA	4	2	2	2,0	–	–	–	2	56	14	4	1
37	Alonso-Almeida, MdM	ESP	4	1	1	4,0	–	–	–	1	1604	68	22	–
38	Aiken, Kirk Damon	USA	4	1	1	4,0	–	–	–	1	233	12	6	–
39	Hur, Y	KOR	3	2	1	1,5	–	–	–	2	120	11	4	–
40	Yim, B. H.	USA	1	2	1	0,5	–	–	–	2	28	7	3	–

*R, ranking; TCES, TPES and HES, total citation, papers and H-index in ESM field; ACP, average citations per author paper in ESM field; Q1: 2002−2004; Q2: 2005−2009; Q3: 2010−2014; Q4: 2015−2019; TC and TP, total citations and papers received by each author (includes papers in other research fields); H, H-index of each author (includes documents in other research fields); TP50 number of papers in the Top 50 list shown in Table 4.*

First, note that the first three indicators (TCES, TPES and HES), consider only the number of citations, papers and the h-index of each author regarding their contributions to the ESM research topic. However, the authors often collaborate and contribute to other areas of research, so we present these same indicators (TC, TP and H) that include these contributions at a general level. Overall, these latter indicators show that several prominent authors have impacted science in general. These include, for example, Funk and Anguera, each with over 2000 citations and 100 papers. However, by focusing only on the field analyzed in this study, Kaplanidou clearly stands out as the most influential and productive author since he has the best indicator outcomes for all the indicators of the analysis, that is, in the indicators of influence (TCES), productivity (TPES) and h-index. However, it is also noted that this same author has not contributed in the last five years (Q4). Other influential authors in this field are Madrigal and Smith, both with more than 98 citations each. Following these authors, please note that the citation indicator falls in the range of 50−60 citations, which is applicable to Filo, Breuer and Hallmann. Several authors also stand out for the significant influence they have achieved with the papers they have published in the last five years (Q4), which has allowed them to position themselves in the top 10 positions of influence. This is the case, for example, for Filo or Funk. In relation to the number of articles per author, no great differences are observed between the authors. However, authors such as Hernández-Mendo and Pérez-López occupy the second position for productivity, with 4 articles published in the last five years. To complement this information and therefore provide a more complete view of the authors who publish in this field, other columns have been included in Table 5 that give general bibliometric information about each author. Since most authors are strongly research oriented, the information presented in these columns represents the productivity and influence that these authors generally have in other fields of research.

When analyzing the evolution over time of publications per author, between the first and second five-year periods (Q1 and Q2, respectively), one author appear who are assumed to have begun to focus on this field of research. In the Q1 period, for example, Madrigal, with his article published in the Journal of Leisure Research, appears in the field. In the Q2 period, Madrigal and other authors such as Kaplanidou and Anthonissen each publish an article. In the third period (Q3), several authors appear, but among them, without a doubt, Kaplanidou stands out with the publication of 5 of his 6 articles. The fourth period (Q4) is the period in which most of the authors appear in the field. Among them, Hernández-Mendo and Pérez-López are the most productive authors of the period.

Another interesting aspect to observe is the productivity of the authors in the productive core of the research field. To this end, Table 6 presents a classification of the 40 main authors in relation to the number of documents they have published in the fifteen most productive journals in the field. Note that the journals presented in the table are ordered from left to right according to their productivity. Likewise, the authors presented in Table 6 are ordered in the same way as those in Table 5.

**TABLE 6:** Total papers authors classified by most productive journals.

R	Name	Country	TCES	TPES	HES	SMR	ESMQ	EM	JST	JSM	IJSMM	C&S	IJSMS	APJMS	CPD	JBR	JSR	S&S	SBP	SBIJ	TP15	OJ
1	Kaplanidou, K	USA	240	6	5	–	1	1	–	1	–	–	2	–	–	–	–	–	–	–	5	1
2	Madrigal, R	USA	126	3	3	–	–	–	–	–	–	–	–	–	–	–	–	–	–	–	0	3
3	Smith, Aaron C. T.	UK	98	2	1	–	–	–	–	–	–	–	–	–	–	–	–	–	–	–	0	2
4	Filo, KR.	AUS	56	3	3	1	–	1	–	1	–	–	–	–	–	–	–	–	–	–	3	0
5	Breuer, C	GER	52	2	2	–	–	–	–	–	–	–	1	–	–	–	–	–	–	–	1	1
6	Hallmann, K	GER	52	2	2	–	–	–	–	–	–	–	1	–	–	–	–	–	–	–	1	1
7	Anthonissen, A	NLD	49	1	1	–	–	–	–	–	–	–	–	–	–	–	–	–	–	–	0	1
8	Funk, DC.	USA	40	2	2	1	–	–	–	1	–	–	–	–	–	–	–	–	–	–	2	0
9	Abosag, I	UK	40	1	1	–	–	–	–	–	–	–	–	–	–	–	–	–	–	–	0	1
10	Doyle, JP	AUS	36	2	2	1	–	–	–	1	–	–	–	–	–	–	–	–	–	–	0	2
11	Calabuig-Moreno, F	ESP	34	2	2	–	–	–	–	–	–	–	–	–	–	1	–	–	–	–	1	1
12	Núñez-Pomar, JM	ESP	34	2	2	–	–	–	–	–	–	–	–	–	–	1	–	–	–	–	1	1
13	Crespo-Hervás, J	ESP	34	2	2	–	–	–	–	–	–	–	–	–	–	1	–	–	–	–	1	1
14	Hernández-Mendo, A	ESP	31	4	4	–	–	–	–	–	–	–	–	–	3	–	–	–	–	–	3	1
15	Pérez-López, R	ESP	31	4	4	–	–	–	–	–	–	–	–	–	3	–	–	–	–	–	3	1
16	Lamont, M	AUS	28	2	1	1	–	–	1	–	–	–	–	–	–	–	–	–	–	–	2	0
17	Anguera-Argilaga, MT	ESP	27	3	3	–	–	–	–	–	–	–	–	–	2	–	–	–	–	–	2	1
18	Morales-Sánchez, V	ESP	27	3	3	–	–	–	–	–	–	–	–	–	2	–	–	–	–	–	2	1
19	Adriaanse, J. A.	AUS	25	1	1	1	–	–	–	–	–	–	–	–	–	–	–	–	–	–	1	0
20	Kim, JW	USA	18	2	2	–	–	–	–	1	–	–	–	–	–	–	–	–	1	–	2	0
21	Kent, A	USA	18	2	1	2	–	–	–	–	–	–	–	–	–	–	–	–	–	–	2	0
22	Swanson, S	UK	18	2	1	2	–	–	–	–	–	–	–	–	–	–	–	–	–	–	2	0
23	Bee, C	USA	14	2	2	–	–	–	–	–	–	–	–	–	–	1	–	–	–	–	1	1
24	Kunkel, T	USA	13	2	2	–	1	–	–	1	–	–	–	–	–	–	–	–	–	–	2	0
25	Ko, Y. J	USA	9	2	2	–	–	–	–	–	–	–	–	–	–	–	–	–	–	–	0	2
26	Mutz, M	CHN	8	2	2	–	–	–	–	–	–	1	–	–	–	–	–	–	–	–	1	1
27	Won, D	USA	8	2	1	–	–	1	–	–	–	–	–	–	–	–	–	–	–	–	1	1
28	Chiu, W	GER	8	2	1	–	–	1	–	–	–	–	–	–	–	–	–	–	–	–	1	1
29	Heere, B	USA	7	3	2	2	–	–	–	–	–	–	–	–	–	–	–	–	–	–	2	1
30	Lee, S	KOR	7	2	2	1	–	–	–	–	–	–	–	–	–	–	–	–	–	–	1	1
31	Tyler, B. D	USA	6	2	1	1	–	–	–	–	–	–	–	–	–	1	–	–	–	–	2	0
32	Apostolopoulou, A	USA	6	1	1	–	–	–	–	–	–	–	–	–	–	–	–	–	–	–	0	1
33	Agha, N	USA	6	1	1	1	–	–	–	–	–	–	–	–	–	–	–	–	–	–	1	0
34	Byon, K	USA	5	3	1	–	1	–	–	–	–	–	–	–	–	–	–	–	–	–	1	2
35	Akhoondnejad, A	NZL	5	1	1	–	–	–	–	–	–	–	–	–	–	–	–	–	–	–	0	1
36	Sato, S	USA	4	2	2	–	–	–	1	–	–	–	–	–	–	–	–	–	–	–	1	1
37	Alonso-Almeida, MdM	ESP	4	1	1	–	–	–	–	–	–	–	–	–	–	–	–	1	–	–	1	0
38	Aiken, K. D	USA	4	1	1	–	–	–	–	–	–	–	–	–	–	1	–	–	–	–	1	0
39	Hur, Y	KOR	3	2	1	–	–	–	–	–	–	–	–	–	–	–	–	–	2	–	2	0
40	Yim, B. H.	USA	1	2	1	–	1	–	–	–	–	–	–	–	–	–	–	–	–	–	1	1

*Journal Abbreviations are available in Table 3. TP15, Amount of paper in major journals; OJ, Other journals available in Table 3. TCES, TPES and HES, Total citations, papers and h-index in ESM Research.*

According to Table 6, most authors have published in one of the journals. The results indicate that Kaplanidou is the most published author in these journals, with 5 articles. Please note that the second place is occupied by Filo, with 3 articles. Hernández-Mendo and Pérez-López continue with 3 articles each, and so on. Similarly, Hernández-Mendo and Pérez-López stand out as the most published authors in CPD, with 3 articles each. In global terms, it is interesting to note that these authors tend to publish their articles in four journals, such as SMR, CPD, JBR and JSM in the same order of importance. However, the ten authors with the most citations in the field generally publish in JSM, IJSMS or SMR among others.

#### The Most Productive and Influential Institutions in ESM Research

Research related to emotions and sport management has attracted the attention of important research groups that usually work in several universities around the world. In recent years, several universities have managed to contribute to the research associated with the management of emotions and sport. Table 7 shows the 40 most productive and influential research institutions in this field.

**TABLE 7:** The most influential and productive institution in ESM research.

R	Organization Name	Country	TCES	TPES	HES	ACP	≥50	≥25	≥10	≥5	Q1	Q2	Q3	Q4
1	University of Florida	USA	192	10	6	19,2	2	1	1	3	–	–	5	5
2	Victoria U. Melbourne	AUS	132	3	3	44,0	1	1	–	–	–	–	2	1
3	University of Oregon	USA	128	4	3	32,0	1	1	1	1	1	1	1	1
4	Auburn University	USA	128	2	2	64,0	1	–	1	–	–	1	–	1
5	RMIT University	AUS	98	2	1	49,0	1	–	–	–	–	–	1	1
6	Temple University	USA	78	7	5	11,1	–	1	3	1	–	–	–	7
7	U. of Minnesota	USA	71	3	2	23,7	1	–	1	–	–	–	1	2
8	University of Windsor	CAN	70	3	2	23,3	1	–	–	–	–	–	1	3
9	Griffith University	AUS	66	5	4	13,2	–	1	2	–	–	–	–	5
10	Indiana University	USA	58	6	3	9,7	–	–	2	–	–	–	1	5
11	La Trobe University	USA	55	4	3	13,8	–	1	1	–	–	–	–	4
12	Florida State University	USA	42	2	2	21,0	–	1	1	–	–	–	1	1
13	University of Valencia	ESP	41	4	3	10,3	–	1	2	–	–	1	–	3
14	Bournemouth University	GBR	40	3	2	13,3	–	1	1	–	–	–	–	3
15	Baylor University	USA	40	2	2	20,0	–	1	1	–	–	–	–	2
16	Universitat de Barcelona	ESP	38	4	4	9,5	–	–	3	–	–	–	1	3
17	Universidad de Malaga	ESP	31	4	4	7,8	–	–	2	–	–	–	–	4
18	Southern Cross University	AUS	28	2	1	14,0	–	1	–	–	–	–	–	2
19	Clemson University	USA	24	2	2	12,0	–	–	1	–	–	–	1	1
20	U. of South Australia	AUS	18	2	2	9,0	–	–	1	1	–	–	1	1
21	Loughborough U.	GBR	18	1	1	18,0	–	–	1	–	–	–	–	1
22	Seoul National University	KOR	17	4	3	4,3	–	–	–	2	–	–	–	4
23	Georgia Southern University	USA	16	3	2	5,3	–	–	–	1	–	–	–	3
24	James Madison University	USA	15	2	1	7,5	–	–	1	–	–	–	–	2
25	U. of Illinois at Urbana-Champaign	USA	13	2	2	6,5	–	–	–	2	–	–	–	2
26	University of Johannesburg	ZAF	11	3	2	3,7	–	–	–	2	–	–	2	1
27	Texas A&M University	USA	11	3	2	3,7	–	–	–	1	–	–	–	3
28	Brock University	CAN	11	2	2	5,5	–	–	–	1	–	–	–	2
29	U. of North Alabama	USA	10	2	2	5,0	–	–	–	1	–	–	–	2
30	Liverpool John Moores U.	GBR	9	2	1	4,5	–	–	–	1	–	–	–	2
31	U. of Massachusetts Amherst	USA	7	2	2	3,5	–	–	–	–	–	–	–	2
32	Oregon State University	USA	7	2	2	3,5	–	–	–	–	–	–	1	1
33	Kookmin University	KOR	7	2	2	3,5	–	–	–	–	–	–	–	2
34	Lithuanian Sports U.	LTU	7	2	1	3,5	–	–	–	1	–	–	–	2
35	U. of South Carolina	USA	6	3	2	2,0	–	–	–	–	–	–	–	3
36	Western Carolina University	USA	6	2	1	3,0	–	–	–	1	–	–	–	2
37	Universiti Teknologi MARA	MAL	5	2	1	2,5	–	–	–	1	–	–	1	1
38	Montclair State University	USA	4	2	2	2,0	–	–	–	–	–	–	–	2
39	Konkuk University	KOR	3	2	1	1,5	–	–	–	–	–	–	–	–
40	U. Europea de Madrid	ESP	2	2	1	1,0	–	–	–	–	–	–	–	2

*R, rank; TCES, TPES and HES, total citation, papers and H-index in ESM field; ACP, average citations per paper from each university in ESM field; Q1: 2002−2004; Q2: 2005−2009; Q3: 2010−2014; Q4: 2015−2019; ≥50, ≥25, ≥10, ≥5 number of paper with more than 50, 25, 10 and 5 citations.*

To achieve a complete perspective of the research carried out in these institutions, TCES and TPES are considered. Similar to the analysis by author, different indicators such as the HES, the citation/paper ratio (PCES *hereinafter*) and citation thresholds are also included. Another interesting issue that has been included in this table is the number of publications per university in relation to the time periods. Please note that these data are presented in periods of five years. Finally, please note that this list is ordered according to TCES, although in case of a tie, TPES indicator will be the one that makes the difference, which in this case is HES.

The results indicate that the University of Florida is the leading institution for research on this topic. Please note that this university has managed to position itself in the last 10 years as the most influential (TCES = 192) and the most productive (TPES = 10) institution in the field. The Victoria University of Melbourne is the second most influential, while Temple University is the second most productive with 7 articles. It should be noted that several universities around the world have begun publishing in the field in the last five years (Q4). As usual, United States universities have a strong presence and therefore lead in research. Note that 50% of the universities are located in the United States. Other countries well represented by their universities are Australia, the United Kingdom and Spain. In short, it can be observed that most of the universities that publish on this topic come from North American, European or Oceanic countries, i.e., those countries that have a fairly developed sport industry and generally have outstanding participation in different sport competitions in the world, such as the Olympic Games. Although there is research coming from universities in other parts of the world, it could be pointed out that the research developed in this topic is strongly influenced by the sport development of the countries.

Another aspect related to universities is the analysis of their productivity as it is related to the core of the research on this topic. Table 8 presents 15 journals that publish more on these topics and therefore can be considered the productive nucleus of the research that has been produced in this field.

**TABLE 8:** Total papers of institutions classified by most productive journals.

R	Name	Country	TCES	TPES	HES	SMR	ESMQ	EM	JST	JSM	IJSMM	C&S	IJSMS	APJMS	CPD	JBR	JSR	S&S	SBP	SBIJ	TP15	OJ
1	University of Florida	USA	192	10	6	–	1	–	–	2	1	–	1	–	–	–	–	–	1	–	6	4
2	Victoria U. Melbourne	AUS	132	3	3	1	–	–	–	–	–	–	–	–	–	–	–	–	–	–	1	2
3	University of Oregon	USA	128	4	3	–	–	–	1	–	–	–	–	–	–	–	–	–	–	–	1	3
4	Auburn University	USA	128	2	2	–	–	–	–	–	–	–	–	–	–	–	–	1	–	–	1	1
5	RMIT University	AUS	98	2	1	2	–	–	–	–	–	–	–	–	–	–	–	–	–	–	–	–
6	Temple University	USA	78	7	5	4	1	–	–	2	–	–	–	–	–	–	–	–	–	–	–	–
7	U. of Minnesota	USA	71	3	2	1	1	–	–	1	–	–	–	–	–	–	–	–	–	–	–	–
8	University of Windsor	CAN	70	3	2	–	–	1	–	–	–	–	–	–	–	–	–	–	–	–	2	1
9	Griffith University	AUS	66	5	4	1	–	1	1	2	–	–	–	–	–	–	–	–	–	–	–	–
10	Indiana University	USA	58	6	3	–	3	–	–	–	–	–	–	–	–	–	–	–	–	–	3	3
11	La Trobe University	USA	55	4	3	1	–	1	–	–	–	–	–	–	–	–	–	–	–	–	2	2
12	Florida State University	USA	42	2	2	–	1	–	–	1	–	–	–	–	–	–	–	–	–	–	–	–
13	University of Valencia	ESP	41	4	3	–	–	–	–	–	–	–	–	–	–	1	–	–	–	–	1	3
14	Bournemouth University	GBR	40	3	2	2	–	–	1	–	–	–	–	–	–	–	–	–	–	–	–	–
15	Baylor University	USA	40	2	2	–	–	–	–	1	–	–	–	–	–	–	–	–	–	–	1	1
16	Universitat de Barcelona	ESP	38	4	4	–	1	–	–	–	–	–	–	–	2	–	–	–	–	–	3	1
17	Universidad de Malaga	ESP	31	4	4	–	–	–	–	–	–	–	–	–	3	–	–	–	–	–	3	1
18	Southern Cross University	AUS	28	2	1	1	–	–	1	–	–	–	–	–	–	–	–	–	–	–	–	–
19	Clemson University	USA	24	2	2	–	–	–	–	–	–	–	–	–	–	–	1	–	–	–	1	1
20	U. of South Australia	AUS	18	2	2	–	–	–	1	–	–	–	–	–	–	–	–	–	–	–	1	1
21	Loughborough U.	GBR	18	1	1	3	–	–	–	–	–	–	–	–	–	–	–	–	–	–	–	–
22	Seoul National University	KOR	17	4	3	1	–	–	–	–	–	–	–	–	–	–	–	–	1	–	2	2
23	Georgia Southern University	USA	16	3	2	1	–	–	1	1	–	–	–	–	–	–	–	–	–	–	–	–
24	James Madison University	USA	15	2	1	–	–	–	–	1	–	1	–	–	–	–	–	–	–	–	–	–
25	U. of Illinois at Urbana-Champaign	USA	13	2	2	1	–	–	–	1	–	–	–	–	–	–	–	–	–	–	–	–
26	University of Johannesburg	ZAF	11	3	2	–	–	–	–	–	–	–	–	–	–	–	–	–	–	–	1	2
27	Texas A&M University	USA	11	3	2	2	1	–	–	–	–	–	–	–	–	–	–	–	–	–	–	–
28	Brock University	CAN	11	2	2	–	–	–	–	–	–	–	–	–	–	–	–	–	–	–	–	2
29	U. of North Alabama	USA	10	2	2	2	–	–	–	–	–	–	–	–	–	–	–	–	–	–	–	–
30	Liverpool John Moores U.	GBR	9	2	1	–	–	–	–	–	–	–	–	–	–	–	–	–	–	–	–	2
31	U. of Massachusetts Amherst	USA	7	2	2	1	–	–	–	–	–	–	–	–	–	1	–	–	–	–	–	–
32	Oregon State University	USA	7	2	2	–	–	–	–	–	–	–	–	–	–	1	–	–	–	–	1	1
33	Kookmin University	KOR	7	2	2	1	–	–	–	–	–	–	–	–	–	–	–	–	–	–	1	1
34	Lithuanian Sports U.	LTU	7	2	1	–	–	–	–	–	–	–	–	–	–	–	–	–	–	–	–	2
35	U. of South Carolina	USA	6	3	2	1	–	–	1	–	–	–	–	–	–	–	–	–	–	–	2	1
36	Western Carolina University	USA	6	2	1	1	–	–	–	–	–	–	–	–	–	1	–	–	–	–	–	–
37	Universiti Teknologi MARA	MAL	5	2	1	–	–	–	–	–	–	–	–	–	–	–	–	–	–	–	–	2
38	Montclair State University	USA	4	2	2	–	–	–	1	–	–	–	–	–	–	–	–	–	–	–	1	1
39	Konkuk University	KOR	3	2	1	–	–	–	–	–	–	–	–	–	–	–	–	–	2	–	–	–
40	U. Europea de Madrid	ESP	2	2	1	–	–	–	–	–	–	–	–	–	–	–	–	–	–	–	–	2

*Journal Abbreviations are available in Table 3. TP15, Amount of paper in major journals; OJ, Oteher journals available in Table 3. TCES, TPES and HES, Total citations, papers and h-index of countries in ESM field.*

In general, 28,6% of the articles produced by these universities have been published in this group of 15 journals. Note that these universities tend to publish in leading journals such as *Sport Management Review*, *Journal of Sport Management* and *European Sport Management Quarterly*, in which they have published 27, 12 and 9 articles, respectively. Of the institutions in this ranking, University of Florida stands out as the most productive in these journals, with 6 articles. Even so, it is expected that these and other universities will start looking for opportunities to publish more in the journals that are considered to be at the core of the research in this topic.

#### Analysis by Country

Finally, to complete the analysis of bibliometric performance, productivity by country is analyzed. This analysis is always interesting, knowing that countries invest in research to foster their development and economic growth. In this study, the analysis is performed to obtain a more complete view of the countries that are paying more attention to this research topic. Please note that researchers can be very mobile internationally, particularly those without language barriers. Therefore, it is likely that these authors may present publications affiliated with different countries. A clear example in this study is that of Kaplanidou, who until 2007 was affiliated with the University of Windsor in Canada and later changed her affiliation to the University of Florida in the United States. In this study, the country declared by the author at the time of publication is taken into account. Table 9 presents a ranking of the top 50 countries in the research topic, which are ordered by their influence (TCES). In the case of a tie, TPES indicator is taken into account, which in this case is HES.

**TABLE 9:** The most productive and influential countries in ESM research.

R	Country	TCES	TPES	HES	ACP	≥100	≥50	≥25	≥10	≥5	Q1	Q2	Q3	Q4
1	United States	982	70	18	14,0	1	6	7	9	10	1	3	16	50
2	Australia	389	21	11	18,5	–	2	4	5	1	–	–	6	15
3	United Kingdom	367	20	8	18,4	1	1	4	1	2	1	4	1	14
4	Germany	184	9	6	20,4	–	1	2	2	2	–	1	3	5
5	Canada	166	7	4	23,7	–	2	1	–	1	1	1	1	4
6	South Korea	125	11	5	11,4	–	1	–	1	3	–	–	–	11
7	Spain	89	12	6	7,4	–	–	1	3	2	–	1	1	10
8	France	65	2	2	32,5	–	1	–	–	1	–	1	–	1
9	Portugal	56	1	1	56,0	–	1	–	–	–	–	–	1	–
10	Netherlands	49	1	1	49,0	–	–	1	–	–	–	1	–	–
11	Malasya	22	4	2	5,5	–	–	–	1	1	–	–	1	3
12	Austria	18	3	2	6,0	–	–	–	1	1	–	–	1	2
13	China	14	3	2	4,7	–	–	–	1	–	–	–	–	3
14	Denmark	13	2	2	6,5	–	–	–	1	–	–	1	1	–
15	Japan	12	2	2	6,0	–	–	–	1	–	–	–	–	2
16	South Africa	11	5	2	2,2	–	–	–	–	1	–	–	2	3
17	Greece	11	3	2	3,7	–	–	–	–	1	–	1	–	2
18	Mexico	10	2	2	5,0	–	–	–	–	1	–	1	–	1
19	Hong Kong	9	3	1	3,0	–	–	–	–	1	–	–	–	3
20	Taiwan	8	3	2	2,7	–	–	–	–	–	–	–	1	2
21	Lithuania	6	2	1	3,0	–	–	–	–	1	–	–	–	2
22	Ecuador	5	1	1	5,0	–	–	–	–	1	–	–	–	1
23	New Zealand	5	1	1	5,0	–	–	–	–	1	–	–	–	1
24	Israel	4	1	1	4,0	–	–	–	–	–	–	–	–	1
25	Italy	2	2	1	1,0	–	–	–	–	–	–	–	–	2
26	Peru	1	1	1	1,0	–	–	–	–	–	–	–	–	1
27	Romania	1	1	1	1,0	–	–	–	–	–	–	–	1	–
28	Slovenia	1	1	1	1,0	–	–	–	–	–	–	–	–	1
29	Sweden	1	1	1	1,0	–	–	–	–	–	–	–	–	1
30	Russia	0	2	0	0,0	–	–	–	–	–	–	–	–	2
31	Argentina	0	1	0	0,0	–	–	–	–	–	–	–	–	1
32	India	0	1	0	0,0	–	–	–	–	–	–	–	–	1
33	Montenegro	0	1	0	0,0	–	–	–	–	–	–	–	–	1
34	Norway	0	1	0	0,0	–	–	–	–	–	–	–	–	1
35	Pakistan	0	1	0	0,0	–	–	–	–	–	–	–	–	1
36	Poland	0	1	0	0,0	–	–	–	–	–	–	–	–	1
37	Singapore	0	1	0	0,0	–	–	–	–	–	–	–	–	1
38	Switzerland	0	1	0	0,0	–	–	–	–	–	–	–	–	1

*R, rank; TCES, TPES and HES, total citation, papers and H-index in ESM field; ACP, average citations per paper from each country in ESM field; ≥100, ≥50, ≥25, ≥10, ≥5 number of paper with more than 100, 50, 25, 10 and 5 citations; Q1: 2002−2004; Q2: 2005- 2009; Q3: 2010−2014; Q4: 2015−2019.*

The data presented clearly show that the United States is the leading country in all dimensions. That is, the United States is the most influential and the most productive country, with 982 citations and 70 papers, respectively. These results are reasonable considering the size of the country and its high investment in Research and Development (R&D). Furthermore, it should be taken into account that an important portion of the universities presented in Table 7 are North American. Please note that the results for the United States are more than double those for the Australia, which has received 389 citations for its 21 published papers. The third country in this ranking is United Kingdom, with 367 citations and 20 papers. Consider that North America leads in productivity and influence with 79 documents totaling 1,158 citations. Europe, while close to North American productivity, has not yet reached the impact of publications produced in this region. Another region that stands out is Oceania, which has achieved a good level of citation for far fewer documents. Asian countries also have a good rate of participation in research on this topic, with South Korea standing out among the top 10 countries. Latin American and African countries, although they appear with some documents, must continue to encourage research in this and other scientific areas.

When the temporal evolution of publications per country is analyzed, it is observed that research on this topic from 20 years ago was concentrated in Anglo-Saxon countries, that is, the United States, the United Kingdom and Canada. The rest of the countries began to show interest in this topic between 2005 and 2014. The last 5 years (2015−2019) represent the period in which almost all the countries began to be interested and to publish on this topic. It is worth noting that some countries are attracting attention due to the rapid growth in their publications. Such is the case of Australia, which without publications in Q1 and Q2, published 21 articles in the following 10 years (Q3 y Q4).

Another aspect that is interesting to analyze is the productivity of the countries as it is related to the core of the research in this topic. For this purpose, the productivity of these countries in the 15 main journals will be taken into account. The results are shown in Table 10.

**TABLE 10:** Total papers of countries classified by most productive journals.

R	Name	SMR	ESMQ	EM	JST	JSM	IJSMM	C&S	IJSMS	APJMS	CPD	JBR	JSR	S&S	SBP	SBIJ	OJ	TP
1	United States	11	6	2	3	6	2	3	2	1	–	2	2	1	2	2	25	70
2	Australia	7	–	3	2	2	–	–	–	1	–	–	–	–	–	1	6	21
3	United Kingdom	5	–	2	1	–	1	–	–	1	–	–	1	–	–	–	9	20
4	Germany	–	–	–	–	2	–	1	2	–	–	–	–	–	–	–	4	9
5	Canada	–	–	1	1	–	1	–	–	–	–	–	–	–	–	1	3	7
6	South Korea	1	–	–	–	–	–	–	1	1	–	–	–	–	3	–	8	11
7	Spain	–	1	–	–	–	–	–	–	–	3	1	–	1	–	–	6	12
8	France	–	–	–	–	–	–	–	–	–	–	–	1	–	–	–	1	2
9	Portugal	–	1	–	–	–	–	–	–	–	–	–	–	–	–	–	0	1
10	Netherlands	–	–	–	–	–	–	–	–	–	–	–	–	–	–	–	1	1
11	Malasya	–	–	–	–	–	–	–	1	–	–	–	–	–	–	1	3	4
12	Austria	–	–	–	–	–	–	–	–	–	–	–	–	1	–	–	2	3
13	China	2	–	–	–	–	–	–	–	–	–	–	–	–	–	–	1	3
14	Denmark	1	–	–	–	–	1	–	–	–	–	–	–	–	–	–	0	2
15	Japan	–	–	–	1	–	–	–	–	–	–	–	–	–	–	–	1	2
16	South Africa	–	–	–	–	–	–	–	–	–	–	–	–	–	–	–	5	5
17	Greece	–	–	–	–	–	1	–	–	–	–	–	–	–	–	1	2	3
18	Mexico	–	–	–	–	–	–	–	–	–	1	–	–	–	–	–	1	2
19	Hong Kong	–	–	1	–	–	–	–	–	1	–	–	–	–	–	–	1	3
20	Taiwan	1	–	–	–	–	–	–	–	–	–	–	–	–	–	–	2	3
21	Lithuania	–	–	–	–	–	–	–	–	–	–	–	–	–	–	–	2	2
22	Ecuador	–	–	–	–	–	–	–	–	–	–	–	–	–	–	–	1	1
23	New Zealand	–	–	–	–	–	–	–	–	–	–	–	–	–	–	–	1	1
24	Israel	–	–	–	–	–	–	–	–	–	–	–	–	–	–	–	1	1
25	Italy	–	–	–	–	–	–	–	–	–	–	–	–	–	–	–	2	2
26	Peru	–	–	–	–	–	–	–	–	–	–	–	–	–	–	–	1	1
27	Romania	–	–	–	–	–	–	–	–	–	–	–	–	–	–	–	1	1
28	Slovenia	–	–	–	–	–	–	–	–	–	–	–	–	–	–	–	1	1
29	Sweden	–	–	–	–	–	–	–	–	–	–	–	–	–	–	–	1	1
30	Russia	–	–	–	–	–	–	–	–	–	–	–	–	–	–	–	2	2
31	Argentina	–	–	–	–	–	–	–	–	–	–	–	–	–	–	–	1	1
32	India	–	–	–	–	–	–	–	–	–	–	–	–	–	–	–	1	1
33	Montenegro	–	–	–	–	–	–	–	–	–	–	–	–	–	–	–	1	1
34	Norway	–	–	–	1	–	–	–	–	–	–	–	–	–	–	–	0	1
35	Pakistan	–	–	–	–	–	–	–	–	–	–	–	–	–	–	–	1	1
36	Poland	–	–	–	–	–	–	–	–	–	–	–	–	–	–	1	1	1
37	Singapore	1	–	–	–	–	–	–	–	–	–	–	–	–	–	–	0	1
38	Switzerland	–	–	–	–	–	–	–	–	–	–	–	–	–	–	–	1	1

*Journal Abbreviations are available in Table 3. TP15, Amount of paper in major journals; OJ, Oteher journals available in Table 3. TPES Total papers of countries in ESM Research.*

The results show that the United States publishes in almost all major journals, except *Cuadernos de Psicología del Deporte*, a journal of Spanish origin, but which also publishes articles in English. Still, the United States outcome are logical given the number of researchers and the productivity within the country. Please note that SMR is the journal in which most American researchers publish. It also seems interesting and anecdotal that the United States publishes the most in ESMQ, even more than all the European countries. Finally, one can observe a general relative presence of the 5 main countries (United States, Australia, United Kingdom, Germany and Canada) in these 15 journals.

### Scientific Mapping Analysis

The following section discusses the graphic mapping of this research topic. This analysis is important because it strengthens and complements the performance analysis. A graphic mapping has the objective of showing the structural aspects of a research field (Gaviria-Marin et al., 2019), in addition to identifying the most representative relationships between the main actors in this research topic. Please note that these analyses are performed on the basis of co-citations (Small, 1973), co-authorships (Peters and van Raan, 1991), bibliographic coupling (Kessler, 1963) and co-words (Callon et al., 1983).

**FIGURE 2:**
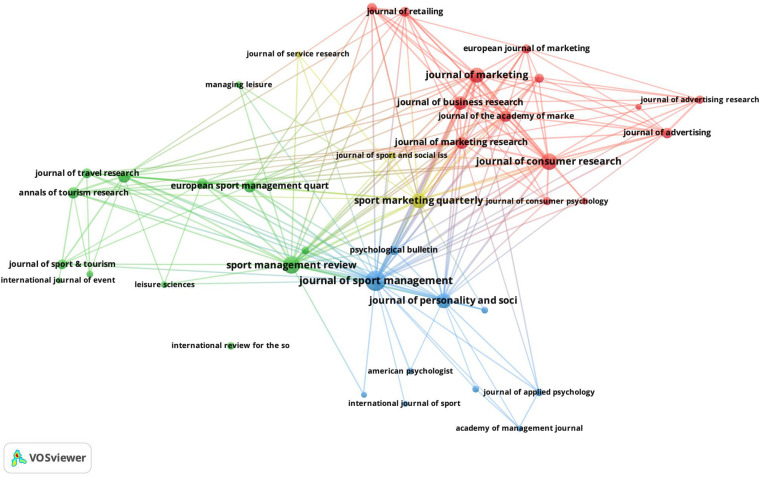
Mapping of co-citation journals.

The graphic mapping of this research topic begins with a graphic analysis of the co-citations or shared journal citations. In other words, this analysis seeks to identify the relationships among journals based on shared citations. Figure 2 presents these relationships, using a threshold of 20 citations and 80 more representative connections.

Please note that in the analysis of the co-citation display, the distance between the journals indicates the relationship of these journals in terms of co-citation. Therefore, it can be seen in Figure 2 that the journals of SMR and JSM are quite related. In addition, given their central location and size, it could be concluded that they are two of the leading journals in this research topic. Please note also that the clusters observed in the figure indicate the relationship between the clustered journals. As is logical, the clustering of these journals also indicates their relationship to specific thematic areas within the research topic. Note, for example, that the red cluster groups journals from the Business and Marketing area mainly. Similar occurs with the journals of the disciplines associated with Tourism and Sport (blue and green clusters). Journals that appear in the periphery are generally emerging in the subject matter and therefore are strongly linked to the main journals that appear in the center of the figure or clusters. Finally, please note that these results are complementary and consistent with the data presented in Table 3.

**FIGURE 3:**
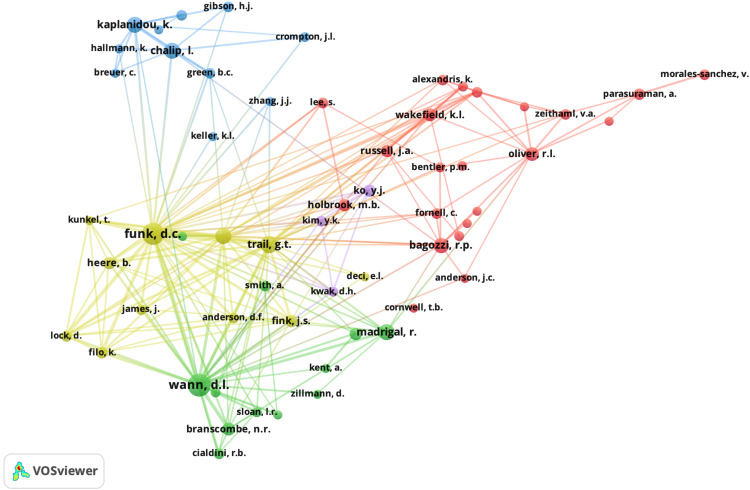
Mapping of co-citation authors.

Another aspect that is analyzed is the co-citation of authors, as presented in Figure 3. Please note that the co-citation of authors represents those most cited among the references analyzed. It also represents the networks or connections that exist between the researchers who publish on this topic. Figure 3 presents these relationships using a threshold of 20 citations and the 100 most-representative connections.

Remember that the co-citation link occurs between two elements that are cited by the same document. In this sense, the visualization of Figure 3 shows several relevant authors who are quite often cited among the references analyzed in this study. Note, for example, that Funk and Wann are relevant authors among the references that have been analyzed. Logically, the closeness of these authors in the graph indicates a strong relationship between them in terms of co-citation. As in the previous figure, please note that several clusters appear that indicate the co-citation relationship between them. An example of this is that Kaplanidou—a relevant author in this study—is co-cited with authors such as Hallmann, Chalip, Gibson, Green, and Breuer, among others. Finally, please note that many of the authors in the figure do not necessarily have to appear among the references analyzed in this study but are rather authors who are co-cited among the references analyzed in this study.

**FIGURE 4:**
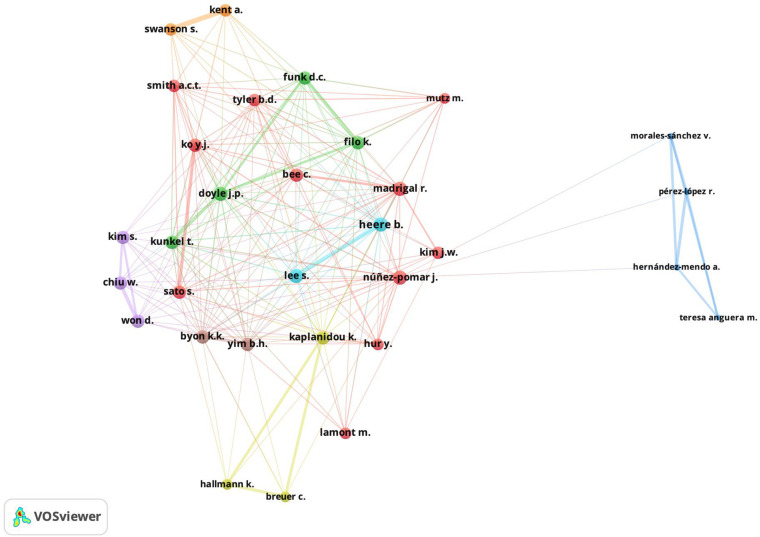
Mapping of authors’ bibliographic coupling.

The most productive authors and how they are connected according to their bibliographic linkages are analyzed below (Kessler, 1963). Please note that this analysis refers to the articles that the researchers have cited in their publications. In addition, please remember that the more references two researchers have in common, the more similar their research is (Ma, 2012). For these purposes, Figure 4 shows the bibliographic linkages of the authors analyzed in this study. This figure shows the results using a threshold of 4 citations and 40 more representative connections.

The figure shows how the authors connect with each other in terms of the common literature. Please note the formation of different clusters that tend to cite the same bibliography. Such is the case of the cluster formed by Kaplanidou, Breuer and Hallmann. It is likely that authors working in the same country or even coauthors of documents will appear in a bibliographic link. An example of this is the cluster formed by Hernández-Mendo, Pérez-López, Morales-Sánchez, and Anguera. This situation usually appears in the analysis of bibliographic linkages, and its explanation is based on the common interests or geographical proximity that researchers from the same country may have.

**FIGURE 5:**
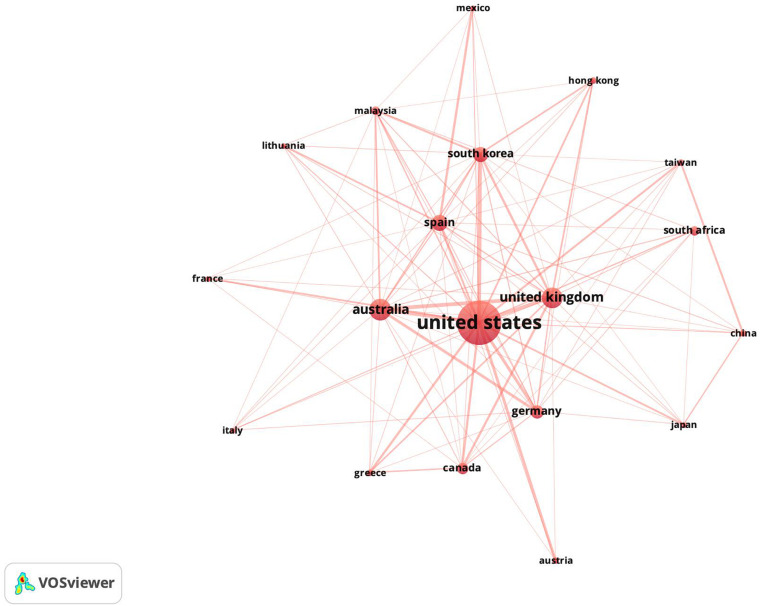
Mapping of countries’ bibliographic coupling.

Following this same type of analysis, the bibliographic linkages of the countries are presented. Therefore, as in the previous explanation, this figure shows how the countries that research this topic are connected bibliographically. Figure 5 shows the bibliographic linkages of the countries considering a threshold of 5 documents and 30 bibliographic linkages.

It is important to highlight that the United States is the leading country in this area of research and therefore appears in the center of the figure. However, countries such as the Australia, United Kingdom, Germany, Canada, Spain and South Korea also appear to be important. Please consider also that this outcome is consistent with what is indicated in Table 9. It is interesting to note that several of the European and Asian countries that appear on the periphery are countries that have begun to publish in the last decade. In the figure, it is also interesting to note that there is no grouping of countries by geographical region. This grouping is normal in literature linkage analyses. This phenomenon can be associated with the youth of the field of study and the clear influence of the countries that appear in the center of the figure.

**FIGURE 6:**
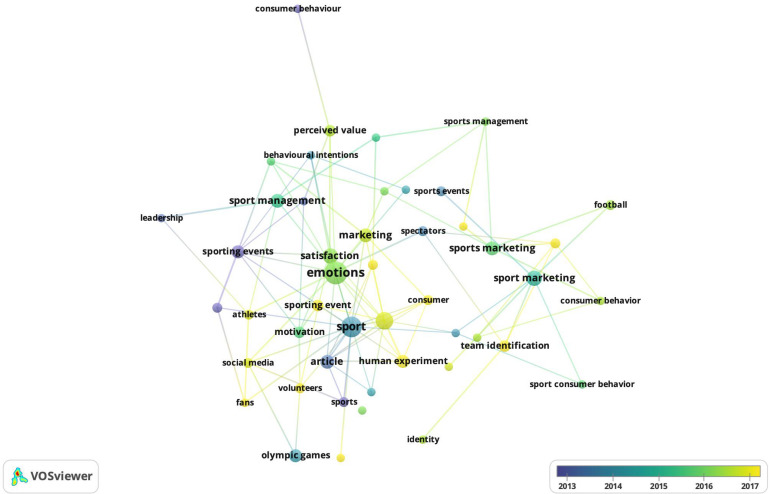
Mapping of co-occurrence of keywords.

Finally, a keyword co-occurrence analysis is developed. This analysis serves to observe the various topics related to the research topic of this study. Figure 6 shows a visualization of the main keywords in the research area using a threshold of four cooccurrences and the seventy most-frequent coincidences. Please note that the visualization is shown according to years. This approach gives an idea of the most recent concepts in relation to the research topic.

**FIGURE 7:**
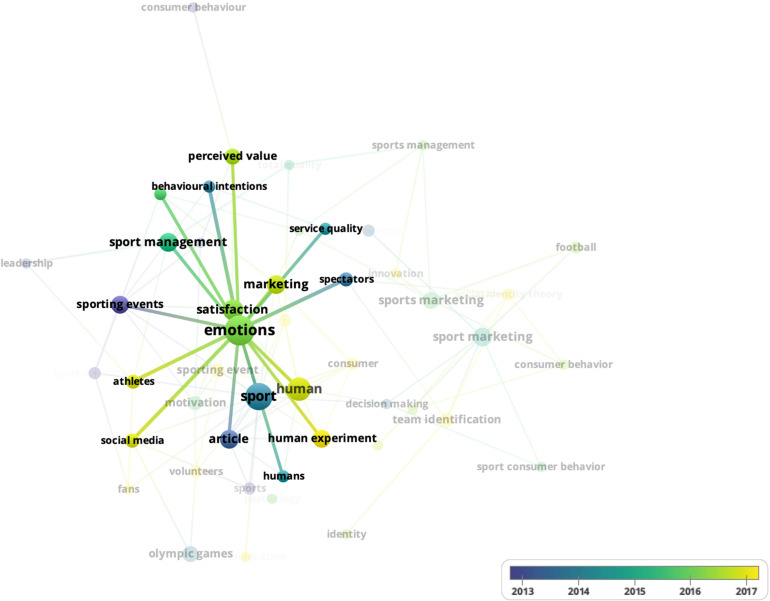
Example of co-occurrence of keywords.

There are several keywords used in this topic. The main keyword used in this research topic is “emotion^∗^.” Please note that other concepts that appear close to the concept of emotions are those of satisfaction, sport, sport events, marketing and sport management, among several others. It is also interesting to note that several concepts have been linked quite frequently in recent years. These can be seen in yellow (Figure 7).

For example, the concepts of marketing, satisfaction or social media have recently been linked in this research topic to the emotion concept. All these words represent the different conceptual frameworks used to explain the intersection of emotions, management and sport.

## Conclusion

Sport management is still a young field of study, which has generated a multidisciplinary academic field linked both to theory and to the needs of the professional world (López-Carril et al., 2019). In this study, a deep bibliometric analysis of the references that are found in the theoretical intersection of emotions and sport management was presented. Over the years, bibliometric studies have integrated a series of techniques and tools that can be complemented to give greater robustness and consistency to the studies (Cobo et al., 2011a). This study is developed based on two bibliometric techniques, namely, a bibliometric performance analysis and a graphic mapping of the knowledge, which have been generated in this research topic.

The overall results indicate that the literature on this research topic has grown significantly in recent years in all scientific disciplines. In this study, the literature was from the areas of business, management, accounting, economics, sociology, psychology, decision sciences and neuroscience have been analyzed. The bibliometric performance analysis performed in this study allows us to realize that the scientific productivity in this topic is led by Anglo-Saxon countries such as the United States, Australia and United Kingdom. The scientific mapping also confirms these results. However, both techniques confirm that of these countries, the most influential country by far is the United States, since it presents the best results of influence, determined as the number of citations received and the h-index. This result is not surprising. In fact, the United States has both universities and authors of great trajectory, who tend to publish in the main journals that give space to this research topic. In addition, this country generally leads scientific research in most scientific areas (Baier-Fuentes et al., 2019). However, in the last five years, several countries have been paying increased attention to this research topic. The cases of Australia, South Korea or Spain for example, is notable and has contributed significantly to the productivity on this topic. The same is true for some Asian countries, such as Korea. However, these countries are still far from the top positions in the field. Other countries, such as those in Latin America or Africa, barely appear with some papers and are therefore encouraged to start expanding their research on this topic.

The United States leads among the countries that are researching this topic, and this situation does not change when institutions are analyzed. In fact, the University of Florida is the most influential and productive institution and therefore lead the research on this topic. As expected, most of the most productive universities are American, which explains the leadership of the United States in this research topic. European universities also have a strong presence, among which Spanish universities such as the University of Valencia, University of Barcelona and the University of Malaga stand out. This outcome should be noted since British universities generally tend to lead scientific research in Europe (based on university rankings).

When analyzing the authors of this topic, we find that Kaplanidou is the most influential and productive author, with good received citations and h-index indicators. Note that this author has 5 documents among the 50 most cited articles in the field. Another important author is Madrigal, who appears as one of the first to focus on this line of research. Nevertheless, it should be noted that several influential authors have appeared in recent years. Some cases are remarkable, such as those of Filo or Funk, with very good indicators of influence reached in the last five years. Spanish authors led by Calabuig-Moreno appear in the last five years with good indicators of influence and productivity. As expected, among the most productive authors, there was an absence of Latin American authors.

The analysis of the journals shows that the literature on this topic has been published in a long list of journals. Logically, given the topic that was analyzed, the journals focused on sport management are the most productive and influential. Among them, the *Sport Management Review* was found to be the most productive and influential journal, with an excellent h-index indicator. Our results also show that the journals of the Business and Marketing areas are important in this topic. The results derived from the mapping of science also confirm that *Sport Management Review* is important in this topic, together with journals such as the *European Sport Management Quarterly*, *Journal of Sport Management*, *Journal of Services Marketing* or *Journal of Sport Management*.

In conclusion, the results of this study may be important for several stakeholders. First, interested researchers would benefit from the relevant information on the main scientific actors who have been publishing on this research topic. Furthermore, knowing this information may be important for profiling and detecting new research ideas (Ferreira, 2018) and may even help in the creation of networks among researchers (Mulet-Forteza et al., 2019); such a network would help to expand and strengthen this striking research topic. Second, the information presented in this study can be used for decision-making both in the political sector and in institutions when deciding to prioritize or fund projects related to this research topic.

However, this paper has several limitations. First, the changing dynamics of science must be taken into account. This implies that various bibliometric performance indicators and the structure of science may change over time. In the last five years, for example, several actors have appeared to publish on this topic, but nothing ensures that they will continue to consolidate and expand their research. Nevertheless, as mentioned, this study intends to present an updated general guide to the research that has been carried out at the intersection of emotions, management and sport. Second, the bibliometric performance indicators are based on the analysis of scientific publications, involving articles, reviews, letters and notes. This implies that several other influential documents may have been excluded from this analysis. Likewise, the use of Scopus as the main source of the references analyzed could also imply the exclusion of other documents. Some authors, such as Jacsó (2008), have pointed out that the loss or exclusion of some references is an endemic problem in bibliographic databases. To this end, future studies should extend or complement this bibliometric analysis to other databases such as WoS, EBSCO, Procuest, among others. Even so, to our knowledge, this study manages to represent quite well the main scientific actors who have contributed to this interesting line of research.

## Data Availability Statement

The original contributions presented in the study are included in the article/Supplementary Material, further inquiries can be directed to the corresponding author.

## Author Contributions

HB-F performed the search and the bibliometric and mapping science analysis describing the methodology and the results. MG-S has conceptualized and critically reviewed the manuscript. MA-D has critically reviewed the manuscript contributing from the theoretical perspective to this special issue. WI-M and VP-E developed part of the bibliometric performance analysis. All authors collaborated in the analysis of the results.

## Conflict of Interest

The authors declare that the research was conducted in the absence of any commercial or financial relationships that could be construed as a potential conflict of interest.

## References

[B1] Addolorato S. Calabuig F. Prado-Gascó V. Gallardo L. García-Unanue J. (2019). Bibliometric analysis of fitness equipment: how scientific focuses affect life-cycle approaches and sustainable ways of development. *Sustainability* 11:5728 10.3390/su11205728

[B2] Agarwal A. Durairajanayagam D. Tatagari S. Esteves S. C. Harlev A. Henkel R. (2016). Bibliometrics: tracking research impact by selecting the appropriate metrics. *Asian j. Androl.* 18 296–309. 10.4103/1008-682X.171582 26806079 PMC4770502

[B3] Alonso S. Cabrerizo F. J. Herrera-Viedma E. Herrera F. (2009). h-Index: a review focused in its variants, computation and standardization for different scientific fields. *J. Informetr.* 3 273–289. 10.1016/j.joi.2009.04.001

[B4] Alonso-Dos Santos M. A. Baeza S. Lizama J. C. (2019). “The intention of attending a sporting event through expectation disconfirmation and the effect of emotions,” in *Integrated Marketing Communications, Strategies, and Tactical Operations in Sports Organizations*, ed. Manuel Alonso D. S. (Pennsylvania: IGI Global), 230–240.

[B5] Alvarado L. (2008). Neuromarketing, ciencia al servicio del conocimiento. *Cuad. Investig.* 2 1–12.

[B6] Baier-Fuentes H. Merigó J. M. Amorós J. E. Gaviria-Marín M. (2019). International entrepreneurship: a bibliometric overview. *Int. Entrep. Manag. J.* 15 385–429. 10.1007/s11365-017-0487-y

[B7] Belfiore P. Iovino S. Tafuri D. (2019). Sport management and educational management: a bibliometric analysis. *Sport Sci.* 12 61–64.

[B8] Benson A. M. Wise N. (2017). *International Sports Volunteering.* London, UK: Routledge.

[B9] Biscaia R. Correia A. Rosado A. Maroco J. Ross S. (2012). The effects of emotions on football spectators’ satisfaction and behavioural intentions. *Eur. Sport Manag. Q.* 12 227–242. 10.1080/16184742.2012.679949

[B10] Bonilla C. A. Merigó J. M. Torres-Abad C. (2015). Economics in Latin America: a bibliometric analysis. *Scientometrics* 105 1239–1252. 10.1007/s11192-015-1747-7

[B11] Broadus R. N. (1987). Toward a definition of “bibliometrics.”. *Scientometrics* 12 373–379. 10.1007/bf02016680

[B12] Calabuig F. Crespo J. Núñez-Pomar J. Valantine I. Staskeviciute I. (2016). Role of perceived value and emotions in the satisfaction and future intentions of spectators in sporting events. *Eng. Econ.* 27 221–229.

[B13] Calabuig F. Prado-Gascó V. Crespo J. Núñez-Pomar J. Añó V. (2015). Spectator emotions: effects on quality, satisfaction, value, and future intentions. *J. Bus. Res.* 68 1445–1449. 10.1016/j.jbusres.2015.01.031

[B14] Calabuig-Moreno F. Gonzalez-Serrano M. H. Alonso-Dos-Santos M. Gómez-Tafalla A. (2020). Entrepreneurial ecosystems, knowledge spillovers, and their embeddedness in the sport field: a bibliometric and content analysis. *Knowl. Manag. Res. Pract.* [Epub ahead of print].

[B15] Callon M. Courtial J. P. Turner W. A. Bauin S. (1983). From translations to problematic networks: an introduction to co-word analysis. *Soc. Sci. Inf.* 22 191–235. 10.1177/053901883022002003

[B16] Campo M. Champely S. Louvet B. Rosnet E. Ferrand C. Pauketat J. V. T. (2019). Group-based emotions: evidence for emotion-performance relationships in team sports. *Res. Q. Exerc. Sport* 90 54–63. 10.1080/02701367.2018.1563274 30707087

[B17] Cancino C. A. Amirbagheri K. Merigó J. M. Dessouky Y. (2019). A bibliometric analysis of supply chain analytical techniques published in computers & industrial engineering. *Comput. Ind. Eng.* 137 106015 10.1016/j.cie.2019.106015

[B18] Cancino C. A. Merigó J. M. Coronado F. C. (2017a). Big names in innovation research: a bibliometric overview. *Curr. Sci.* 113 1507–1518.

[B19] Cancino C. A. Merigó J. M. Coronado F. Dessouky Y. Dessouky M. (2017b). Forty years of computers & industrial engineering: a bibliometric analysis. *Comput. Ind. Eng.* 113 614–629. 10.1016/j.cie.2017.08.033

[B20] Carneiro M. J. Eusébio C. Caldeira A. Santos A. C. (2019). The influence of eventscape on emotions, satisfaction and loyalty: the case of re-enactment events. *Int. J. Hosp. Manag.* 82 112–124. 10.1016/j.ijhm.2019.03.025

[B21] Chen C. (2006). CiteSpace II: detecting and visualizing emerging trends and transient patterns in scientific literature. *J. Am. Soc. Inf. Sci. Technol.* 57 359–377. 10.1002/asi.20317

[B22] Ciomaga B. (2013). Sport management: a bibliometric study on central themes and trends. *Eur. Sport Manag. Q.* 13 557–578. 10.1080/16184742.2013.838283

[B23] Cobo M. J. López-Herrera A. G. Herrera-Viedma E. Herrera F. (2010). An approach for detecting, quantifying, and visualizing the evolution of a research field: a practical application to the fuzzy sets theory field. *J. Informetr.* 5 146–166. 10.1016/j.joi.2010.10.002

[B24] Cobo M. J. López-Herrera A. G. Herrera-Viedma E. Herrera F. (2011a). An approach for detecting, quantifying, and visualizing the evolution of a research field: a practical application to the fuzzy sets theory field. *J. Informetr.* 5 146–166.

[B25] Cobo M. J. López-Herrera A. G. Herrera-Viedma E. Herrera F. (2011b). Science mapping software tools: review, analysis, and cooperative study among tools. *J. Am. Soc. Inf. Sci. Technol.* 62 1382–1402. 10.1002/asi.21525

[B26] Dickson T. J. Benson A. M. Terwiel F. A. (2014). Mega-event volunteers, similar or different? Vancouver 2010 vs London 2012. *Int. J. Event Festiv. Manag.* 5 164–179. 10.1108/ijefm-07-2013-0019

[B27] Ding J. Liu C. Kandonga G. A. (2020). Exploring the limitations of the h-index and h-type indexes in measuring the research performance of authors. *Scientometrics* 122 1303–1322. 10.1007/s11192-020-03364-1

[B28] Dubé L. Menon K. (2000). Multiple roles of consumption emotions in post-purchase satisfaction with extended service transactions. *Int. J. Serv. Ind. Manag.* 11 287–304. 10.1108/09564230010340788

[B29] Egghe L. (2006). Theory and practise of the g-index. *Scientometrics* 69 131–152. 10.1007/s11192-006-0144-7

[B30] Egghe L. (2010). The hirsch index and related impact measures. *Annu. Rev. Inf. Sci. Technol.* 44 65–114. 10.1002/aris.2010.1440440109

[B31] Ferreira F. A. F. (2018). Mapping the field of arts-based management: bibliographic coupling and co-citation analyses. *J. Bus. Res.* 85 348–357. 10.1016/j.jbusres.2017.03.026

[B32] Foroughi B. Mohammad Shah K. A. Ramayah T. Iranmanesh M. (2019). The effects of peripheral service quality on spectators’ emotions and behavioural intentions. *Int. J. Sport. Mark. Spons.* 20 495–515. 10.1108/ijsms-08-2018-0082

[B33] Funk D. Lock D. Karg A. Pritchard M. (2016). Sport consumer behavior research: improving our game. *J. Sport Manag.* 30 113–116. 10.1123/jsm.2016-0028

[B34] Gaviria-Marin M. Merigó J. M. Baier-Fuentes H. (2019). Knowledge management: a global examination based on bibliometric analysis. *Technol. Forecast. Soc. Chang.* 140 194–220. 10.1016/j.techfore.2018.07.006

[B35] Gaviria-Marin M. Merigo J. M. Popa S. (2018). Twenty years of the journal of knowledge management: a bibliometric analysis. *J. Knowl. Manag.* 22 1655–1687. 10.1108/jkm-10-2017-0497

[B36] Gellweiler S. Fletcher T. Wise N. (2019). Exploring experiences and emotions sport event volunteers associate with ‘role exit.’. *Int. Rev. Sociol. Sport* 54 495–511. 10.1177/1012690217732533

[B37] González-Serrano M. H. Jones P. Llanos-Contrera O. (2019). An overview of sport entrepreneurship field: a bibliometric analysis of the articles published in the Web of Science. *Sport Soc.* 23 296–314. 10.1080/17430437.2019.1607307

[B38] Güntert S. T. Neufeind M. Wehner T. (2015). Motives for event volunteering: extending the functional approach. *Nonprof. Volunt. Sect. Q.* 44 686–707. 10.1177/0899764014527797

[B39] Hirsch J. E. (2005). An index to quantify an individual’s scientific research output. *Proc. Natl. Acad. Sci. U.S.A.* 102 16569–16572. 10.1073/pnas.0507655102 16275915 PMC1283832

[B40] Jacsó P. (2008). The pros and cons of computing the h-index using Web of Science. *Online Inf. Rev.* 32 673–688. 10.1108/14684520810914043

[B41] Jang W. Byon K. K. Yim B. H. (2019). Sportscape, emotion, and behavioral intention: a case of the big four US-based major sport leagues. *Eur. Sport Manag. Q.* 20 321–343.

[B42] Jin B. H. Liang L. M. Rousseau R. Egghe L. (2007). The R- and AR-indices: complementing the h-index. *Chinese Sci. Bull.* 52 855–863. 10.1007/s11434-007-0145-9

[B43] Jones M. V. (2003). Controlling emotions in sport. *Sport Psychol.* 17 471–486. 10.1123/tsp.17.4.471

[B44] Kessler M. M. (1963). Bibliographic coupling between scientific papers. *Am. Doc.* 14 10–25. 10.1002/asi.5090140103

[B45] Kleinginna P. R. Kleinginna A. M. (1981). A categorized list of emotion definitions, with suggestions for a consensual definition. *Motiv. Emot.* 5 345–379. 10.1007/bf00992553

[B46] Koenig-Lewis N. Palmer A. (2014). The effects of anticipatory emotions on service satisfaction and behavioral intention. *J. Serv. Mark.* 28 437–451. 10.1108/jsm-09-2013-0244

[B47] Laengle S. Merigó J. M. Miranda J. Słowí Nski R. Bomze I. Borgonovo E. (2017). Forty years of the European journal of operational research: a bibliometric overview. *Eur. J. Oper. Res.* 262 803–816. 10.1016/j.ejor.2017.04.027

[B48] Lewis M. Haviland-Jones J. M. Barrett L. F. (2008). *Handbook of Emotions. The Philosophy of Emotions.* New Yok, NY: The Guilford Press.

[B49] López-Carril S. Villamón M. Sanz V. A. (2019). Conceptualisation of Social Media: opportunities for Sport Management. *Retos* 36 468–473.

[B50] Ma R. (2012). Author bibliographic coupling analysis: a test based on a Chinese academic database. *J. Informetr.* 6 532–542. 10.1016/j.joi.2012.04.006

[B51] Malchrowicz-Mośko E. Chlebosz K. (2019). Sport spectator consumption and sustainable management of sport event tourism; fan motivation in high performance sport and non-elite sport. A case study of horseback riding and running: a comparative analysis. *Sustainability* 11:2178 10.3390/su11072178

[B52] Martin D. O’neill M. Hubbard S. Palmer A. (2008). The role of emotion in explaining consumer satisfaction and future behavioural intention. *J. Serv. Mark.* 22 224–236. 10.1108/08876040810871183

[B53] Martínez-López F. J. Merigó J. M. Valenzuela-Fernández L. Nicolás C. (2018). Fifty years of the European Journal of Marketing: a bibliometric analysis. *Eur. J. Mark.* 52 439–468. 10.1108/ejm-11-2017-0853

[B54] Meho L. I. Yang K. (2007). Impact of data sources on citation counts and rankings of LIS faculty: web of science versus scopus and google scholar. *J. Am. Soc. Inf. Sci. Technol.* 58 2105–2125. 10.1002/asi.20677

[B55] Merigó J. M. Gil-Lafuente A. M. Yager R. R. (2015). An overview of fuzzy research with bibliometric indicators. *Appl. Soft Comput.* 27 420–433. 10.1016/j.asoc.2014.10.035

[B56] Mongeon P. Paul-Hus A. (2016). The journal coverage of Web of Science and Scopus: a comparative analysis. *Scientometrics* 106 213–228. 10.1007/s11192-015-1765-5

[B57] Muhuri P. K. Shukla A. K. Abraham A. (2019). Industry 4.0: a bibliometric analysis and detailed overview. *Eng. Appl. Artif. Intell.* 78 218–235. 10.1016/j.engappai.2018.11.007

[B58] Mulet-Forteza C. Genovart-Balaguer J. Mauleon-Mendez E. Merigó J. M. (2019). A bibliometric research in the tourism, leisure and hospitality fields. *J. Bus. Res.* 101 819–827. 10.1016/j.jbusres.2018.12.002

[B59] Noyons E. C. M. Moed H. F. Luwel M. (1999). Combining mapping and citation analysis for evaluative bibliometric purposes: a bibliometric study. *J. Am. Soc. Inf. Sci.* 50 115–131. 10.1002/(sici)1097-4571(1999)50:2<115::aid-asi3>3.0.co;2-j

[B60] Ong D. L. T. Yap W. X. (2017). The impact of fitness center servicescape on individual behavior: the mediating role of emotional response. *J. Glob. Sport Manag.* 2 128–142. 10.1080/24704067.2017.1314177

[B61] Pedragosa V. Biscaia R. Correia A. (2015). The role of emotions on consumers’ satisfaction within the fitness context. *Motriz. Rev. Educ. Fis.* 21 116–124. 10.1590/s1980-65742015000200002

[B62] Persson O. Danell R. Wiborg Schneider J. (2009). “How to use Bibexcel for various types of bibliometric analysis,” in *Celebrating Scholarly Communication Studies: A Festschrift for Olle Persson at his 60th Birthday*, eds Åström F. Danell R. Larsen B. Schneider J. (Leuven: International Society for Scientometrics and Infometrics), 9–24.

[B63] Peters H. P. F. van Raan A. F. (1991). Structuring scientific activities by co-author analysis: an exercise on a university faculty level. *Scientometrics* 20 235–255. 10.1007/bf02018157

[B64] Podsakoff P. M. MacKenzie S. B. Podsakoff N. P. Bachrach D. G. (2008). Scholarly influence in the field of management: a bibliometric analysis of the determinants of university and author impact in the management literature in the past quarter century. *J. Manage.* 34 641–720. 10.1177/0149206308319533

[B65] Porter A. L. Cunningham S. W. (2005). *Tech Mining: Exploiting New Technologies for Competitive Advantage.* Hoboken, NJ: John Wiley & Sons Inc.

[B66] Prathap G. (2010). Is there a place for a mock h-index? *Scientometrics* 84 153–165. 10.1007/s11192-009-0066-2

[B67] Puig N. (2012). Emociones en el deporte y sociología. *Int. J. Sport Sci.* 8 106–108.

[B68] Ramos-Rodrígue A. R. Ruíz-Navarro J. (2004). Changes in the intellectual structure of strategic management research: a bibliometric study of the Strategic Management Journal, 1980-2000. *Strateg. Manag. J.* 25 981–1004. 10.1002/smj.397

[B69] Rialp A. Merigó J. M. Cancino C. A. Urbano D. (2019). Twenty-five years (1992–2016) of the international business review: a bibliometric overview. *Int. Bus. Rev.* 28:101587 10.1016/j.ibusrev.2019.101587

[B70] Rodriguez-Pomeda J. Casani F. del Mar Alonso-Almeida M. (2017). Emotions’ management within the Real Madrid football club business model. *Soccer Soc.* 18 431–444. 10.1080/14660970.2014.980736

[B71] Scherer K. (1987). Toward a dynamic theory of emotion. The component process of affective states. *Cogn. Emot.* 1 1–72.

[B72] Schmitt B. (1999). Experiential marketing. *J. Mark. Manag.* 15 53–67. 10.1362/026725799784870496 30847903

[B73] Silla A. Calabuig F. Añó V. (2014). Emotions, satisfaction and future intentions of guided sport activities users’ of a sport center. *J. Sport. Econ. Manag.* 4 22–38.

[B74] Small H. (1973). Co-citation in the scientific literature: a new measure of the relationship between two documents. *J. Am. Soc. Inf. Sci.* 24 265–269. 10.1002/asi.4630240406

[B75] Terán-Yépez E. Marín-Carrillo G. M. del Casado-Belmonte M. P. de las Capobianco-Uriarte M. M. (2020). Sustainable entrepreneurship: review of its evolution and new trends. *J. Clean. Prod.* 252:119742 10.1016/j.jclepro.2019.119742

[B76] Valenzuela L. M. Merigó J. M. Johnston W. J. Nicolas C. Jaramillo J. F. (2017). Thirty years of the journal of business & industrial marketing: a bibliometric analysis. *J. Bus. Ind. Mark.* 32 1–17.

[B77] Vallerand R. J. Blanchard C. M. (2000). “The study of emotion in sport and exercise,” in *Emotions in Sport*, ed. Hanin Y. L. (Champaign, IL: Human Kinetics Publishers Inc.), 3–37.

[B78] van Eck N. J. Waltman L. (2010). Software survey: VOSviewer, a computer program for bibliometric mapping. *Scientometrics* 84 523–538. 10.1007/s11192-009-0146-3 20585380 PMC2883932

[B79] van Kleef G. A. Cheshin A. Koning L. F. Wolf S. A. (2019). Emotional games: how coaches’ emotional expressions shape players’ emotions, inferences, and team performance. *Psychol. Sport Exerc.* 41 1–11. 10.1016/j.psychsport.2018.11.004

[B80] Wise J. A. (1999). The ecological approach to text visualization. *J. Am. Soc. Inf. Sci.* 50 1224–1233. 10.1002/(sici)1097-4571(1999)50:13<1224::aid-asi8>3.0.co;2-4

[B81] Wolfe R. A. Weick K. E. Usher J. M. Terborg J. R. Poppo L. Murrell A. J. (2005). Sport and organizational studies exploring synergy. *J. Manag. Inq.* 14 182–210. 10.1177/1056492605275245

[B82] Yan Z. Wu Q. Li X. (2016). Do Hirsch-type indices behave the same in assessing single publications? An empirical study of 29 bibliometric indicators. *Scientometrics* 109 1815–1833. 10.1007/s11192-016-2147-3

[B83] Ye F. Y. Leydesdorff L. (2014). The “academic trace” of the performance matrix: a mathematical synthesis of the h-index and the integrated impact indicator (I3). *J. Assoc. Inf. Sci. Technol.* 65 742–750. 10.1002/asi.23075

[B84] Zhang C. T. (2013). The h’-index, effectively improving the h-index based on the citation distribution. *PLoS One* 8:e59912. 10.1371/journal.pone.0059912 23565174 PMC3614896

